# Snow algae exhibit diverse motile behaviors and thermal responses

**DOI:** 10.1128/mbio.02954-24

**Published:** 2025-04-01

**Authors:** Alexandre Détain, Hirono Suzuki, René H. Wijffels, Nathalie Leborgne-Castel, Chris J. Hulatt

**Affiliations:** 1 Faculty of Biosciences and Aquaculture, Nord University 168026, Bodø, Norway; 2 Bioprocess Engineering, AlgaePARC, Wageningen University 593528 , Wageningen, the Netherlands; 3 Agroécologie, INRAE, Institut Agro, Université Bourgogne Europe 27011, Dijon, France; University of Georgia, Athens, Georgia, USA; University of Georgia, Athens, Georgia, USA

**Keywords:** snow algae, motility, swimming, cilia, temperature

## Abstract

Snow algal blooms influence snow and glacier melt dynamics, yet the mechanisms involved in community assemblage, development, and dispersal are not well understood. While microbial swimming behavior contributes significantly to the productivity and organization of aquatic and terrestrial microbiomes, the potential impact of algal cell motility in melting snow on the formation of visible, large-scale surface bloom patterns is largely unknown. Here, using video tracking and phototaxis experiments of unique isolates, we evaluated the motility of diverse snow algal taxa from green, red, and golden colored snow blooms in response to light and thermal gradients. We show that many species are efficient cryophilic microswimmers with speed thermal optima below 10°C although taxa with cryotolerant swimming traits were also identified. The significant motility of snow algae at low temperatures, a result of specialized adaptations, supports the importance of active movement in the life histories of algae inhabiting snow meltwater. However, diversity in swimming performance and behavior reveal a range of evolutionary outcomes and sensitivity of motile life stages to dynamic environments.

**IMPORTANCE:**

Swimming motility is a fundamental mechanism that controls the assembly, structure, and productivity of microbiomes across diverse environments and is highly sensitive to temperature. Especially, the role of cell swimming activity in algal bloom formation at the very low temperatures of snowmelt has been hypothesized, but not studied. By examining the movement patterns of snow algae and modeling the thermal response curves of swimming speed, the data reveal the key role of active cell movement that may have further important impacts on the microbial ecology and melt rates of snow and ice in polar and alpine regions.

## INTRODUCTION

Snow-covered terrestrial surfaces including seasonal snow and glaciers account for, at their maximal extent, nearly 50% of the global land area (1). Diverse microbial communities proliferate in snow, driving unique ecological processes with additional bio-optical impacts that influence snowmelt and the mass balance of glaciers (2, 3). During the summer in polar and alpine areas, algal blooms cause strikingly colored snow, which ranges from bright green to orange, golden, and red, depending on the species and physiological stage (4). Algae are the major primary producers in snow and ice ecosystems, and they support a wealth of associated microbial diversity ranging from bacteria and fungi to predatory protists and viruses (5–7). Snow algal blooms are often large enough to be detected by satellite and can be tracked to follow their dynamics (8, 9). The biogeography of blooms is thought to be driven by complex interplay between ecological and physical variables, including the altitude, climate, seasonal melt dynamics, underlying soil structure, dispersal, and biotic factors (10–12). Significantly, many species of snow algae are motile, yet it is not known how the swimming behavior of individual cells might impact the formation of blooms.

Here, we investigated how the microscale motility of snow algae, in response to light and temperature, may enable population movement and bloom formation in cold environments, as it does with phytoplankton in the formation of harmful algal blooms (13) or in enhancing oceanic primary production (14). However, snow algae occupy more extreme and dynamic terrestrial environments than their ocean counterparts, experience low temperatures with freeze-thaw cycles, snow cover, dark to very bright light levels, and often display complex life cycles including biciliate or “flagellate” motile stages as well as diverse non-motile cell morphologies (15–18). Several studies have discussed that non-motile cell stages rest beneath the snow during winter and rely on cellular motility to emerge, swimming upward among summer meltwater where they potentially undergo mating, propagate, and eventually transform to the orange or red colored aplanospore cells comprising watermelon snow (16–22). Bischoff (11) supports this hypothesis with an observation of cultivated species migrating 5 cm upward within 1 day in microcosm-maintained summer melting snow from the Swiss Alps, and a recent field study in Alaska suggests that active cell movement through the snowpack is responsible for about 65% of the bloom intensity (22). Diel vertical migration of microbes comprising a small green bloom, which includes ciliated snow algae, has also been reported within a snowpack in northern Japan (23).

At a minimum, the presence of thin water films, as found on damp soil or around the surfaces of melting ice crystals, is required for microswimming. In polar and alpine regions, the availability of liquid water films is linked to the snowpack thaw dynamics during spring and summer (11, 12, 24), which varies with the climate and altitude. Microbial motility is guided by gradients of competing stimuli in the environment, including chemicals (chemotaxis) (25), temperature (thermotaxis) (26), and light (phototaxis) (13) that are actively sensed by diverse membrane receptors and sensors (27), plus morphological features that passively orientate cells by gravity (gravitaxis) (28). Together, these evolved directional controls shape the organization of aquatic microbiomes, leading to the formation of hyper-productive microbial communities (13, 14, 29). In snow, both passive gravitactic orientation and active light-directed phototaxis might contribute to the vertical migration of cells and shape bloom formation at the surface, and the low temperatures may select for novel adaptive and evolutionary mechanisms that support energetic ciliary movement in the cold (30–33).

Our overall aim was to investigate the motility of novel snow-inhabiting algal taxa across thermal gradients, from the low temperatures of snowmelt to the higher surface soil temperatures found later in summer. Our research addresses several important initial questions: (i) How is snow algal motility and movement impacted by temperature? (ii) What is the underlying diversity in swimming behavior, phototaxis, and cell dispersal potential among snow algae? (iii) Can the data provide insight into the likely ecological impacts of movement on snow algal blooms and microbiome development? After benchmarking the experimental set-up, a series of motility and phototaxis measurements were conducted using novel isolates to uncover the diversity in snow algal movement.

## RESULTS

### Experimental temperature control modulates motility

**Fig 1:**
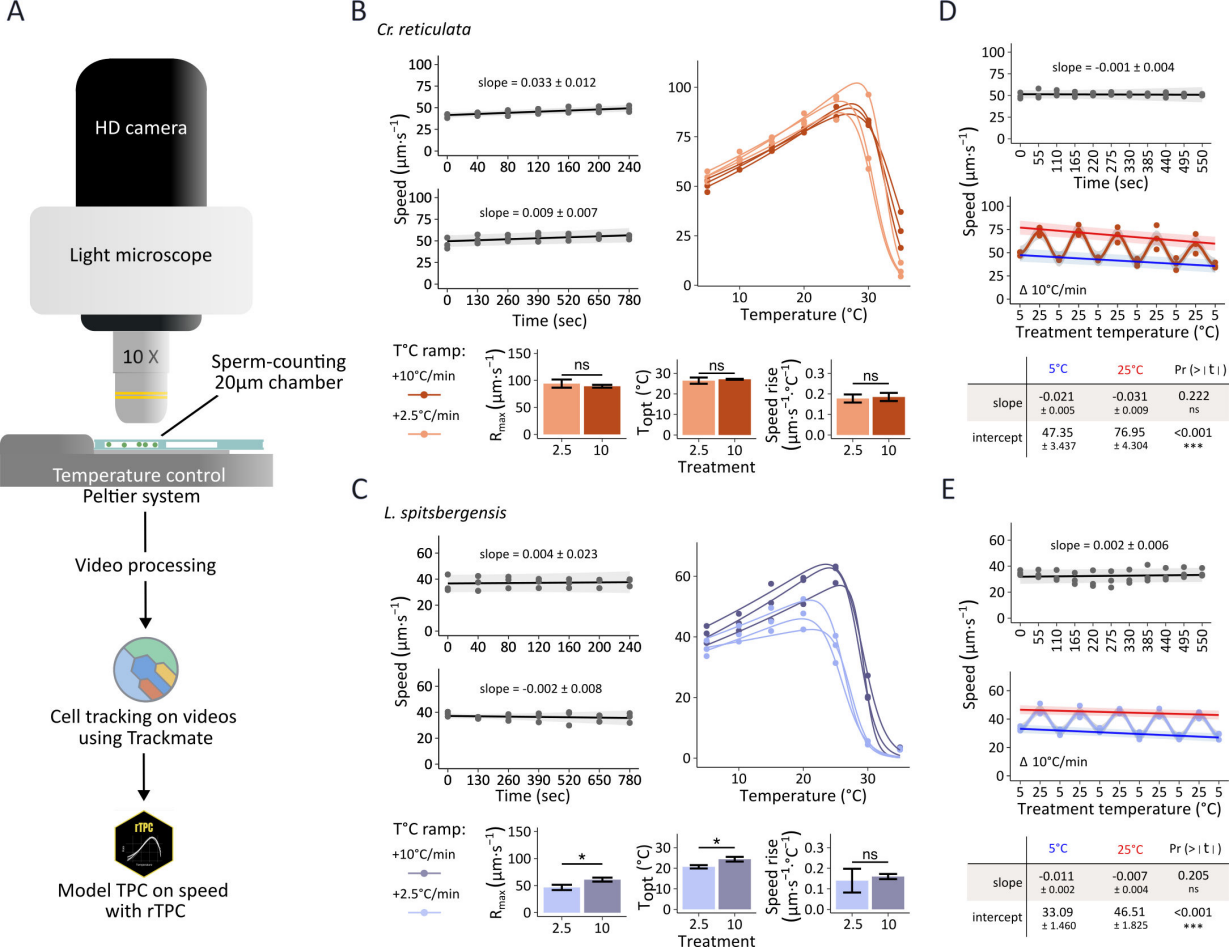
Benchmarking real-time temperature-control system to measure microbial motility. (A) Experimental set-up for measuring microbe swimming speed under temperature control. (B, C) Effect of constant temperature and increasing temperature gradients on the swimming speed of *Chloromonas reticulata* (B) and *Limnomonas spitsbergensis* (C) including panels representing controls (gray) held under constant temperature of 5°C, and panels under increasing temperature from 5 to 35°C at a rate of 10°C⋅min ^-1^ (dark color) and 2.5°C⋅min ^-1^ (light color). To test the stability of swimming speed over time, a linear mixed effects model was fitted (*n* = 3) and the slope with 95% confidence intervals is shown (shaded area). Under increasing temperature gradients, thermal performance curves (TPCs) were fitted using the Pawar model, and means (±sd, *n* = 3) of associated parameters are presented including *R*_max_, *T*_opt_, and the rise in swimming speed rise with increasing temperature (slope). Statistical tests (*t*-test, *n* = 3, *P* < 0.05) compare the effect of slow and fast temperature ramps on the TPC parameters. (D, E) Changes in the swimming speed of *Cr. reticulata* (D) and *L. spitsbergensis* (E) under oscillating temperature Δ 10°C⋅min^−1^ between 5°C and 25°C. Top panels show first the controls (gray) under constant 5°C temperature, while panels below (middle) in color display the variation in swimming speed under dynamic temperature. Linear mixed effects models were fitted to describe the speed change during temperature oscillation cycles between 5°C steps (blue) at 25°C steps (red). Intercepts and slopes (± standard error) for each temperature resulting from the model are presented in the table (bottom) and the interaction between them is described as Pr (> | *t* |).

The experimental apparatus for recording motility was first benchmarked by testing the effect of temperature control on microalgal swimming speed using two highly motile species, the cryotolerant *Chloromonas reticulata* strain ARK-S12-19 (15) and the cryophilic *Limnomonas spitsbergensis* strain CCCryo_020-99_CH (34). Algal cells were loaded into a specialized microscope slide, a “sperm-counting chamber,” that was mounted on the thermally controlled stage of a microscope fitted with a video camera, and movies recorded the swimming dynamics of populations (Fig. 1A). First, we established that in control treatments operated at a constant temperature of 5°C, cell swimming speeds did not significantly change over experimental durations of 240–780 s (Fig. 1B through E). Next, we showed that experimental temperature ramps from 5 to 35°C induced immediate changes in the swimming speed of both species, compared to controls maintained at constant temperature (*P* < 0.05, *n* = 3, Fig. 1B and C). The swimming responses under temperature ramps were characterized by acceleration up to a thermal limit, followed by the abrupt inactivation of motility at 25–30°C. For both species, the temperature ramp rate (+2.5°C⋅min^−1^ vs +10°C⋅min^−1^) did not significantly affect the rise in swimming speed to optimal temperature although the slower +2.5°C⋅min^−1^ ramp resulted in earlier fatigue of the cryophilic *L. spitsbergensis* that was characterized by a shift in *T*_opt_ to lower temperature (*P* = 0.013) and also a lower maximum swimming speed or *R*_max_ (*P* = 0.018) (Fig. 1C). Finally, to characterize the real-time swimming response to dynamic slide temperature control, we evaluated swimming speed under rapid temperature oscillations from 5 to 25°C at a rate of Δ10°C⋅min^−1^. Slide thermal oscillation caused repeated changes in cell speed, with limited evidence of fatigue as shown by weak negative slopes at the upper and lower temperature bounds (Fig. 1D and E). In total, our benchmarking data collectively demonstrate stable experimental control under constant temperature, and the ability to sensitively measure changes in swimming speed in response to thermal variation.

### Snow algae isolate morphology and phototaxis

**Fig 2:**
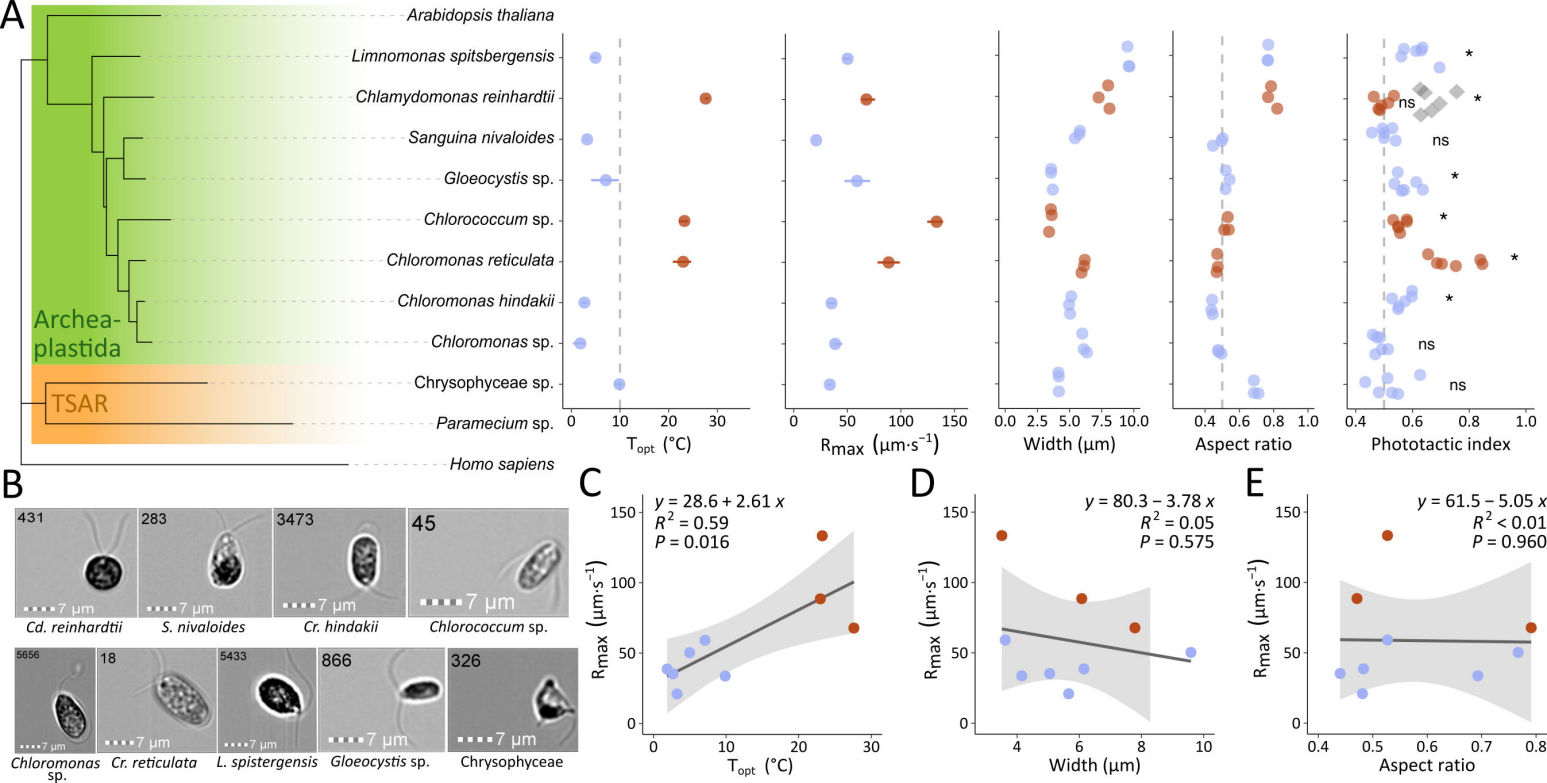
Phylogeny, morphology, and swimming characteristics of the nine studied taxa. (A) *Left*, Phylogeny based on 18S rDNA sequences between the nine species in the present study. Eight studied taxa belong to the Archaeplastida and specifically the Chlamydomonadales of the core Chlorophytes. The plant model Arabidopsis, a ciliate (*Paramecium* sp.) and human (*Homo sapiens*) are included for reference. A single strain is a novel chrysophyte from the TSAR supergroup (Telonemia, Stramenopiles, Alveolates, Rhizaria). *Right,* features of the studied species including *T*_opt_ the optimal swimming temperature (°C) and *R*_max_ the maximum swimming speed (µm⋅s^−1^) based on thermal performance curves, the cell width (µm) and cell aspect ratio of swimming cells measured by imaging flow cytometry (IFC), and phototactic index at 2.5°C, where gray diamonds represent the positive control *Cd. reinhardtii* at 20°C. Phototaxis behavior was assessed by a *t*-test and significant result marked with asterisks (*n* = 6, mean ≠ 0.5; *P* < 0.05). (B) Representative images (Fig. S6) of typical swimming cells of each taxa from IFC, digits indicate cell ID. (C–E) Linear correlations between the maximum swimming speed (*R*_max_) and (C) optimal temperature (*T*_opt_,°C), (D) cell width (µm) and (E) cell aspect ratio, with 95% confidence intervals in a shaded area. Blue and brown color code differentiates taxa with cryophilic behavior (*T*_opt_ < 10°C, blue) from cryotolerant/mesophilic behavior (brown, *T*_opt_ > 20°C) observed in this study.

The study was subsequently conducted using eight snow algal taxa, including new isolates from Arctic Norway and additional strains from diverse thermal backgrounds (Fig. 2; Table 1). The model alga *Chlamydomonas reinhardtii*, which has known mesophilic swimming characteristics, was included as a ninth and control strain. Seven of the snow algae belonged to the Chlorophyceae, each of which have green motile cell stages with two symmetrical cilia (Fig. 2A and B). The eighth snow isolate was a novel *Hydrurus*-like strain (Chrysophyceae) from the TSAR supergroup (Telonemia, Stramenopiles, Alveolates, Rhizaria), which has two different, asymmetric cilia. The Chlorophycean taxa are found widespread in green, orange, or red snow blooms, while Chrysophyceae are associated with golden colored snow. Two of our isolates are of special interest since they form pigmented cell stages in the field; *Chloromonas hindakii* forms orange resting cells with ornamented thick cell wall (35), while *Sanguina nivaloides* is renowned as the cosmopolitan species causing vivid red or “watermelon snow” comprising small, round, smooth-walled astaxanthin-rich cells (36).

**TABLE 1:** Strain information and features

Species	Strain number	Location	Type bloom sampled	Visible eyespot	Phototaxis	Key features
*Limnomonas spitsbergensis*	CCCryo 020-99_CH	Spitsbergen (Svalbard)	Green/red	Yes	Yes	Cells green biciliates with cryophilic traits; DUF3494 ice-binding proteins in the genome (34)
*Sanguina nivaloides*	ARK-S31-22	Nordland (Norway)	Red	No	No	Main alga causing “watermelon snow” globally; red resting cells or “cysts” containing astaxanthin; green biciliates in culture (36)
*Gloeocystis* sp.	ARK-S23-21	Nordland (Norway)	Red	Yes	Yes	Extracellular polymeric substances, EPS exudates (see Fig. S1)
*Chlorococcum* sp.	ARK-S22-20	Nordland (Norway)	Red	Yes	Yes	Isolated from snow but more commonly associated with soil
*Chloromonas reticulata*	ARK-S12-19	Nordland (Norway)	Red	Yes	Yes	Cosmopolitan in snow blooms, but not primary bloom forming species (15)
*Chloromonas hindakii*	CCCryo 531-19	High Tatras Mountain (Poland)	Orange	No	Yes	Hard and ornamented cell wall with orange pigmentation in blooms; green biciliates in culture (35)
*Chloromonas* sp.	ARK-S34-22	Nordland (Norway)	Red	No	No	Novel strain from the low-temperature snow-algae clade (4)
Chrysophyceae sp.	ARK-S30-22	Nordland (Norway)	Golden	Yes	No	Two asymmetric hairy cilia and golden pigmentation including fucoxanthin and chlorophyll *c* (4)
*Chlamydomonas reinhardtii*	CCAP 11-32C	MA (USA)	–^*a*^	Yes	Yes	Not a snow-associated species, sampled from a freshwater lake; included as a mesophilic control species with known motility characteristics

^*a*^ –, not applicable.

The swimming cells have an ovoid cell morphology with aspect ratios around 0.5, except for Chrysophyceae sp., *Cd. reinhardtii*, and *L. spitsbergensis* which were more rounded, with aspect ratios between 0.7 and 0.8 (Fig. 2A and B). *Chlamydomonas reinhardtii* and *L. spitsbergensis* were each also the largest cells, measuring 8 and 9 µm in mean width respectively. *Gloeocystis* sp., *Chlorococcum* sp., and Chrysophyceae sp. were the smallest cells, measuring 3.5–4 µm in width. Morphologically, microscopic examination showed that six of the taxa had a visible eyespot though we could not observe this feature in the cells of *Chloromonas* sp., *Cr. hindakii*, or *S. nivaloides*.

In relation to sensing light with an eyespot, phototaxis Petri dish assays revealed whether cell populations could respond by migrating toward or away from light at low temperature (2.5°C). Significant, positive phototactic behavior was observed for five snow algae including *L. spitsbergensis*, *Gloeocystis* sp., *Chlorococcum* sp., *Cr. reticulata,* and *Cr. hindakii* (*P* ≤ 0.045, *n* = 6, *t*-test, Fig. 2A). The control strain *Cd. reinhardtii* was positively phototactic at 20°C (adj. *P* < 0.01, *n* = 6, *t*-test), but inactivated at 2.5°C (adj. *P* = 1, *n* = 6, *t*-test), highlighting the specialized light-directed behavior of many snow-inhabiting species at low temperature. Non-phototactic responses were recorded for Chrysophyceae sp., *S. nivaloides,* and *Chloromonas* sp. (adj. *P* ≥ 0.778, *n* = 6, *t*-test).

### Temperature dependence of snow algal motility

**Fig 3:**
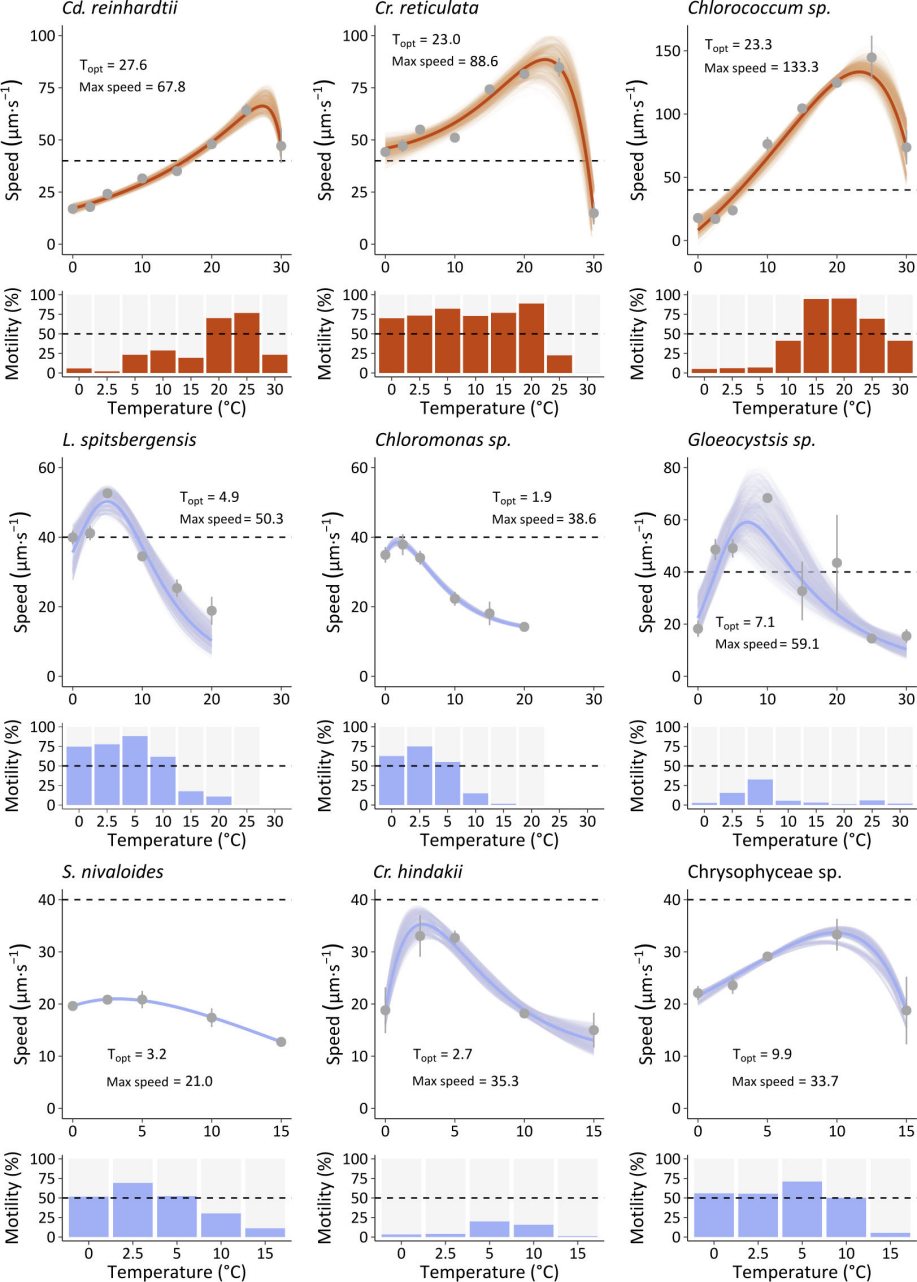
Thermal performance curves of swimming speeds and corresponding proportion of motile cells. Thermal performance curves (TPCs, upper panels) were modeled based on the speed from the “motile” cell population (bar chart, lower panels) at each temperature (°C). TPCs were modeled using the weighted bootstrapping method with residual resampling and (37) model (7 taxa), except for *Limnomonas spitsbergensis* and *Gloeocystsis* sp. that were modeled with the (38) model. Gray dots represent mean (±sd, *n* = 3) of measured swimming speed. Dashed lines mark 40 µm⋅s^−1^ for all TPC plots, and 0.5 proportion for all motility plots. *T*_opt_ (°C) and the maximum swimming speed (*R*_max_) (µm⋅s^−1^) from models are indicated. Blue and brown color code differentiates taxa with observed cryophilic behavior (blue) from cryotolerant/mesophilic behavior (brown).

Computer-assisted tracking of cells incubated at different temperatures for 48 h revealed strong temperature-dependent motility responses, with substantial heterogeneity between species (Fig. 3; Fig. S2). For all species, both the proportion of motile cells within a population and the swimming speed (µm⋅s^−1^) varied with the temperature. Indeed, the swimming speed of all strains increased with temperature to a thermal optimum (*T*_opt_), above which motility was inactivated, and the response profiles were accurately modeled by fitting thermal performance curves (TPCs) (Fig. 3). The mesophilic control strain *Cd. reinhardtii* showed the highest proportion of motile cells in the population at 25°C, with predicted *R*_max_ at 27.6°C, and was inactivated at higher temperatures of around 30°C. Comparable mesophilic thermal responses were observed for two of the eight snow algal taxa, which had *T*_opt_ of 23.0 and 23.3°C for *Cr. reticulata* and *Chlorococcum* sp., respectively. *Chlorococcum* sp. notably recorded the fastest swimming speeds of 145 µm⋅s^−1^. However, each behaved very differently at low temperatures from 0 to 5°C, where *Cr. reticulata* maintained populations with a high proportion of motile cells and moderate swimming speeds of about 44–55 µm⋅s^−1^, but the majority of *Chlorococcum* sp. cells did not move, and those showing motility moved at low speeds of around 20 µm⋅s^−1^. At temperatures of 5°C and below, *Chlorococcum* sp. populations were characterized by large palmelloid-like cells, but when gently compressed under the cover slip, a few motile cells emerged (Fig. S3). In contrast, six snow algal taxa showed *T*_opt_ below 10°C, of which four species *L. spitsbergensis*, *Chloromonas* sp., Chrysophyceae sp., and *S. nivaloides* were largely comprised (>50% population) of actively moving cells at temperatures from 0 to 5°C. On the other hand, both *Gloeocystis* sp. and *Cr. hindakii* were weakly motile at 0 and 2.5°C with no more than 16% of the cells actively moving, and were most active at 5°C with only 33% and 20% motile cells, respectively. At higher temperatures of 15°C and above, all these six taxa displaying *T*_opt_ below 10°C showed very weak motility, represented by a low proportion (<18%) of motile cells moving at low speeds.

Because biological rates scale with temperature, the relation between *T*_opt_ (°C) and maximum swimming speed (µm⋅s^−1^) across the nine taxa was examined. A positive linear correlation (*R*^2^ = 0.59, *P* = 0.016) was identified, showing that *R*_max_ increased with *T*_opt_ at a rate of 2.61 µm⋅s^−1^⋅°C^−1^ (Fig. 2C). Unicellular algae that measure up to a few tens of microns in size have low Reynolds numbers and experience primarily viscous forces (39), so the cell size and morphology substantially influence drag during movement. However, for the nine taxa that span a cell width range of 3.4–9.7 µm, there was no detectable correlation between the cell width and *R*_max_ (*R*^2^ = 0.05, *P* = 0.575), nor between the cell aspect ratio and *R*_max_ (*R*^2^ <0.01, *P* = 0.960).

**Fig 4:**
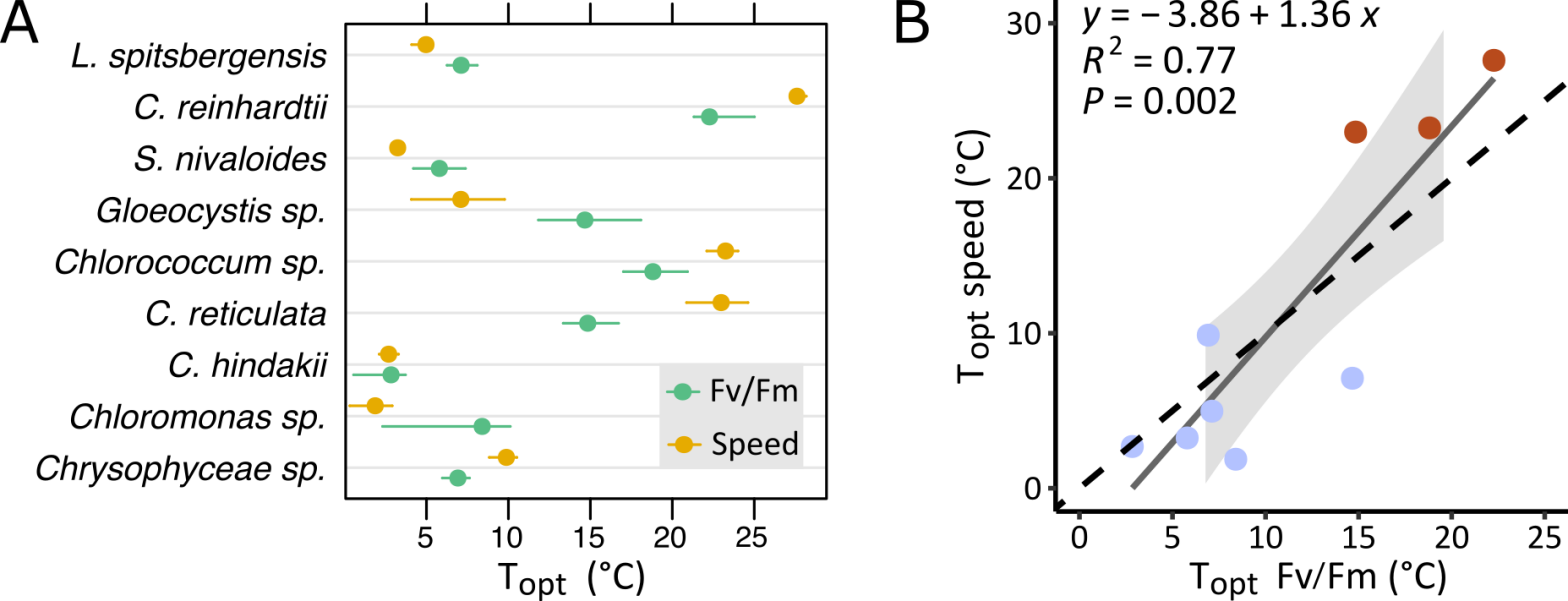
Temperature optimum (*T*_opt_) of swimming speed and photosynthesis. (A) Comparison of the *T*_opt_ (with 95% confidence intervals) from thermal performance curves (TPCs) of swimming speed (yellow) with photosynthetic efficiency Fv/Fm (green), as presented in Fig. 3; Fig. S7. (B) Linear regression model of the *T*_opt_ swimming speed and *T*_opt_ Fv/Fm (solid line) compared with a slope of 1.0 (dashed line) across nine taxa. The 95% confidence intervals in gray include a slope between 0.72 and 2.00.

Alongside motility measurements, the thermal responses of photosynthetic efficiency were evaluated by measuring Fv/Fm at each temperature, and the patterns were modeled with the same TPCs as for the swimming speed (Fig. S4). Thermal ranges based on chlorophyll fluorescence measurements were broadly concordant with motility and swimming speed measurements. For example, *Cr. hindakii*, *S. nivaloides,* and Chrysophyceae sp., that had upper swimming limits of 15°C, were not photosynthetically active above this temperature either. Likewise, the upper thermal limits of swimming and chlorophyll fluorescence of *L. spitsbergensis* and *Chloromonas* sp. were comparable, with no measurable activity above 20°C. To test the association of swimming speed and photosynthetic thermal traits, the *T*_opt_ Fv/Fm and *T*_opt_ swimming speed were compared (Fig. 4). The *T*_opt_ of swimming speed and *T*_opt_ of Fv/Fm of individual strains were offset by 0.15–8.14°C (Fig. 4A), but the differences were species-specific, and across all taxa, the *T*_opt_ swimming speed and *T*_opt_ Fv/Fm were approximately proportional (Fig. 4B).

### Spatial dispersal scales with temperature

**Fig 5:**
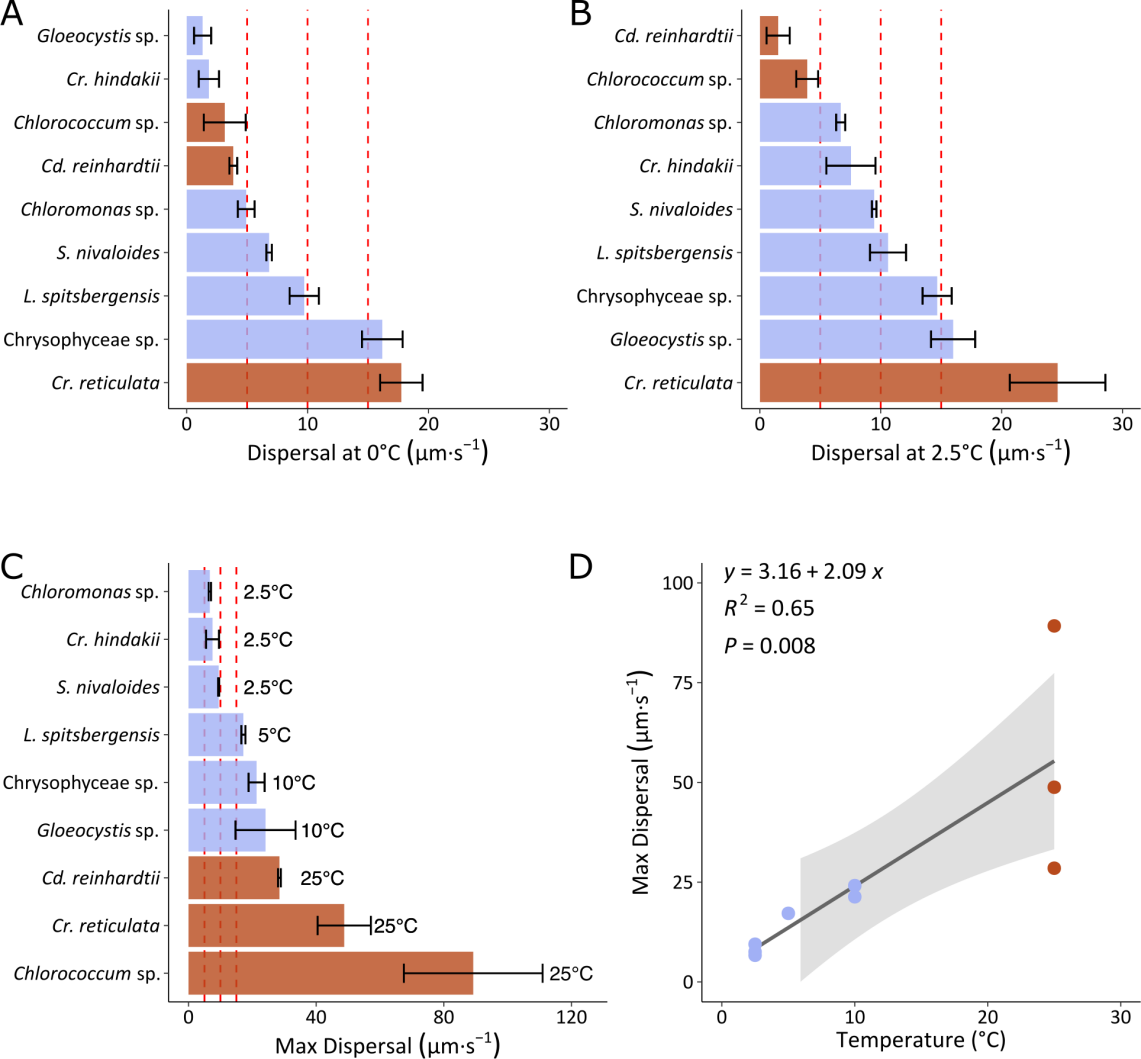
Dispersal of snow algae. Dispersal (µm⋅s^−1^) is calculated from the mean speed multiplied by the confinement ratio. Dispersal at (A) 0°C and (B) 2.5°C. (C) Max Dispersal measured at the corresponding optimal temperature. Values represent means and standard deviations of three replicate cultures. Species are ordered from the lowest to the highest dispersal in (A–C). (D) Linear correlation (95% confidence intervals in shaded area) between Max Dispersal and corresponding temperatures. Blue and brown color code differentiates taxa with cryophilic behavior (*T*_opt_ < 10°C, blue) from cryotolerant/mesophilic behavior (*T*_opt_ > 20°C, brown) observed in this study.

In addition to the absolute speed of swimming, the dispersal and net migration of cells in the environment is determined by the combination of directional changes and 3D swimming patterns. Thus, in addition to thermal effects on outright speed, the distances swum by cells over 10 s video recordings were characterized using the parameters “total distance traveled,” “maximum distance traveled,” and the “confinement ratio” (Fig. S2A; Table S1). While the total distance traveled over 10 s is directly related to the average speed, the maximum distance traveled between two points during this time is the result of cell horizontal dispersal radius (Fig. S2). To describe dispersal (µm⋅s^−1^), average speeds were adjusted based on the calculated confinement ratio (Fig. 5). We first present dispersal estimates at temperatures of 0 and 2.5°C that are relevant to snowmelt (Fig. 5A and B). At 0°C, highest dispersals were observed for *Cr. reticulata* and Chrysophyceae sp. (>15 µm⋅s^−1^ or 1.29 m⋅d^−1^) followed by *L. spitsbergensis* and *S. nivaloides*. At 2.5°C, dispersal was higher, with *Cr. reticulata* showing again the highest rates of net migration, followed by *Gloeocystis* sp. and Chrysophyceae sp. There was a positive linear relationship between maximum dispersal and the temperature (Fig. 5C and D, *R*^2^ = 0.65, *P* = 0.008) so that rates of spatial displacement also scale with temperature.

## DISCUSSION

The role of motility in structuring snow algal blooms remains poorly understood and experimental evidence that swimming behavior could shape the formation of blooms, and, thus, the ecology and melt rates of globally important snow and ice systems, has remained speculative. Here, we recorded the motility and swimming characteristics of diverse snow algae, including our own novel isolates found in green, red, and golden blooms and determined their thermal responses. We showed that, apart from outright speed, there are significant changes in the proportion of motile cells, their movement patterns, and responses to light and temperature that potentially drive species ecological success, control microscale organization, and contribute to bloom formation.

Temperature is a key environmental variable that determines the overall rates of biological processes including photosynthesis and respiration (40), yet surprisingly few studies have exclusively addressed how the movement of diverse protists responds to different thermal regimes. The swimming speed of snow algae responds to rising temperature and thermal oscillations in real-time, and incubations of 48 h across wide thermal ranges lead to changes in cellular motility that can be accurately modeled with conventional TPCs (Fig. 1 and 3). Indeed, the flagellar and ciliary activity of microbes is well known to be temperature dependent and, at least empirically, the flagellar rotation of bacteria and ciliary beating rates of eukaryotes can be approximated by the classical Arrhenius model (30). However, microbial swimming performance is not only dependent on the physiological rates of enzyme kinetics, but also on physical forces including drag, which is related to body size at low Reynolds numbers, and particularly the liquid viscosity, which co-varies with the temperature (41). Although body size controls drag, we did not find any impact of cell size on the swimming speed at *T*_opt_ (Fig. 2D and E), potentially because it is compensated by other evolutionary adaptations such as ciliary length and beat characteristics (42), plus features that also scale with size, such as the total energy (ATP) available to power movement (43). In the model *Cd. reinhardtii*, cell swimming speed and ciliary beat frequency have been shown to respond to temperature in a thermotactic environment (26), and evidence suggests that movement of cilia in response to temperature is not exclusively regulated by physiological rates. For example, cilia involved in motility are regulated by a pool of transient receptor potential (TRP) channels (44), including TRP2 that controls ciliary beat activation and thermotaxis in *Cd. reinhardtii* (32). Thus, the swimming speed of microeukaryotes broadly follows standard thermal performance kinetics that are modified by a variety of biophysical constraints and evolved sensory control systems.

The heterogeneity of *T*_opt_ and *R*_max_, together with variation in the proportion of motile cells, illustrates a range of evolutionary and adaptive outcomes among different snow algal taxa. In terms of the abundance of motile cells within a population, energy availability and sensitivity to temperature are key variables that control the life cycle, morphology and physiology of cells, and consequently the motility (45, 46). The Chlamydomonadalean taxa responded to temperature by adjusting their population structure of actively moving versus deciliated and recruiting cells. This is the result of altered biological rates and thermal stress that define the allocation for cellular growth and repair, together with operating the ciliary machinery, a response that has been observed in *Cd. reinhardtii* in its natural environment (33). The photosynthetic performances Fv/Fm, which are sensitive to temperature and especially to heat stress (47–49), showed comparable thermal ranges and optimal temperatures to the swimming speed across taxa (Fig. 4). The metabolic rates of different cellular processes, including photosynthesis and respiration, co-evolve within taxa (50) although the temperature sensitivity and thermal optima of different metabolic pathways vary (51). Considering the energy cost required to fuel ciliary movement (43) and the potential fitness benefits of motility, investigating related bioenergetic traits including mitochondrial activity, photosynthetic carbon fixation, and the cell energy budget are needed.

Thermal performance curves showed major differences among species, including two clusters of thermal responses (Fig. 3). The first cluster comprised six snow taxa presenting cryophilic swimming traits with *T*_opt_ below 10°C, which is consistent with the growth pattern previously described for many species of snow algae (4, 52). The second cluster of three taxa, including the control *Cd. reinhardtii*, displayed mesophilic swimming traits with *T*_opt_ above 20°C. Broadly, the *R*_max_ swimming speed scaled with *T*_opt_ (Fig. 2C), showing that although snow algae have specifically adapted their ciliary activity to perform at the very cold temperatures of snowmelt, their overall *R*_max_ remains lower (20.9–59.1 µm⋅s^−1^) than those of mesophilic taxa with higher *T*_opt_ (67.8–133.3 µm⋅s^−1^). These results are concordant with the “hotter-is-better” thermal adaptation scenario (53) that describes how, despite adaptive processes, thermodynamic constraints ultimately limit maximum rates of biological performance.

To provide more perspective into the ecological relevance of motility, we examined the cell dispersal under diffuse lightning conditions, and also the phototactic response, which is the sensitivity and movement of populations with respect to light (Fig. 2A and 5; Fig. S2). The swimming path of biciliate cells is defined by a succession of loop and straight-line trajectories, as well as the detail of the helicoidal pattern (42, 54), and influenced by signaling events (e.g., Ca^2+^ signaling) caused by environmental stimuli and internal physiological status that control cilia coordination and beat frequency (32, 55, 56). In parallel with swimming speed, the trajectories and swimming patterns varied between species (Fig. S5) and with temperature. At 0 and 2.5°C, species with high proportions of motile cells were *Cr. reticulata*, Chrysophyceae sp., *L. spitsbergensis,* and *S. nivaloides* (Fig. 5). However, under dim and directional green light, *Chloromonas* sp., Chrysophyceae sp., and *S. nivaloides* were not phototactic. *Sanguina nivaloides* is renowned as the key species causing red “watermelon snow” blooms in polar, alpine, and glacial areas around the world (57). Our data, therefore, raise the question of whether light-directed motility plays a major role in the formation of red snow blooms, and instead whether other features, e.g., gravitaxis that orientate cilia upward and consequently cells to swim up may be important in the potential vertical movement of cells through melting snow. “Aberrant” or non-phototactic behavior has been observed for *Chlamydomonas priscuii* from a dark, saline, ice-covered lake in Antarctica and highlights the heterogeneity in light-sensing apparatus among Chlamydomonadalean algae (58). The loss of visible stigma and non-phototactic orientation has been hypothesized to provide an ecological advantage against excessive light (59) that, in concert with the preservation of ciliary motility, implicates roles of competing yet unexplored sensory systems associated with, for example, chemotaxis (60–63), thermal navigation (32), and potentially sexual reproduction (21, 64) among the Chlamydomonadales. The green light used in this study penetrates relatively deeply into snow (16) and is known to control phototaxis (65), yet the light wavelength and intensity may also affect the magnitude and direction of cellular migration, and our results invite further studies on light response and sensing in diverse non-model algae.

Most species examined here are adapted to swim at very low temperatures relevant to melting snow, and the short-term dispersal rates scale up to between half a meter and more than a meter per day for the four species with the highest motility. Such movement rates might not be realized in the dynamic fluid conditions found among melting snow and ice crystals in the field, but the data indicate the potential of motility to shape the emergence of snow algal communities. During the summer, polar and alpine snowfields undergo protracted melt periods of weeks (16) to several months, and Roussel et al. (12) recently observed that at least 46 days of continuous melting are needed to establish visible surface blooms. Indeed, the expression of motile cell stages, moderate swimming speeds, and modest dispersal rates recorded in our study remain broadly compatible with field observations by Rea and Dial (22), who report active vertical migration of algae within at least a meter and a half of snowpack.

The results highlight the sensitivity of snow algal motility, and protists in general, to rising temperatures. Under climate warming, the increasingly early onset of snowmelt and consequent prolonged snow-free periods during polar and alpine summers will expose snow algal communities to warmer soil environments for longer periods of the year (66). Because snow algal communities are organized along geographic and altitudinal gradients (10), future snow blooms may include changes in community structure, range shifts, or adaptive evolution, with further effects on their associated microbiomes. The evolutionary responses of algal motility to long-term changes in the thermal environment are not known although evidence from wider studies indicates that adaptive processes play a key role, such as adjusting rates of temperature-dependent biological processes (67).

In conclusion, our experiments provide new insights into the thermal responses of algae that are able to swim, disperse, and migrate at temperatures close to that of melting snow. Although such cold environmental conditions may ultimately limit maximum physiological rates and incur an energetic cost for cells, the data support a key role for motility in shaping primary production and algal blooms in high alpine and polar environments. The study also highlights the thermal sensitivity and responsiveness of microbial motility and swimming traits among diverse taxa, a key energetic feature that is often neglected, yet in a warming world could potentially experience selective pressure leading to altered phenotypes, reshape bloom communities, or change the biogeographic distribution of snow algae.

## MATERIALS AND METHODS

### Cultivation and identification

*Chlamydomonas reinhardtii* 11-32C was acquired from the Culture Collection of Algae and Protozoa (CCAP, Oban UK, www.ccap.ac.uk) and included as a mesophilic control or reference strain. *Limnomonas spitsbergensis* (CCCryo 020-99_CH) and *Chloromonas hindakii* (CCCryo 531-19) were obtained from the Culture Collection of Cryophilic algae (CCCryo, Fraunhofer Institute, Potsdam Germany, cccryo.fraunhofer.de). Strain CCCryo 020-99_CH was isolated from melting snow on Spitsbergen, Svalbard, in the Arctic, while strain CCCryo 531-19 was isolated from the high Tatra mountains in Poland. A further six strains were isolated from red snowfields in Nordland, northern Norway: *Chloromonas reticulata* (ARK-S12-19), *Chloromonas* sp. (ARK-S34-22), *Chlorococcum* sp. (ARK-S22-20), *Gloeocystis* sp. (ARK-S23-21), Chrysophyceae sp. (ARK-S30-22), and *Sanguina nivaloides* (ARK-S31-22). The selected strains are representative of diverse snow-inhabiting taxa with global dispersal and large-scale bloom impact. Cultures were maintained in 250 mL Erlenmeyer flasks in a temperature-controlled incubator fitted with cool-white fluorescent lights (Termaks AS, Norway) at 5°C (20°C for *C. reinhardtii*) and illuminated at approximately 40 µmol⋅m^−2^⋅s^−1^ photons photosynthetically active radiation (PAR). *Limnomonas spitsbergensis* and *Cd. reinhardtii* were grown in Tris-Acetate-Phosphate (TAP) medium (68), while the remaining seven strains were grown in Bold’s Basal Medium (BBM) with double nitrate (2N-BBM) concentration (69). The pH was adjusted to 6.5 with HCl for 2N-BBM and acetic acid for TAP medium. All cultures were replicated into new flasks 4 days before the experiment at an optical density (OD) 680 nm of 0.40–0.60 and readjusted to an OD_680_ of 0.25 to start the experimental cultivation.

To construct the phylogeny, the DNA of unique isolates was extracted from pelleted cells using E.Z.N.A. HP Plant DNA Kit (Omega Bio-tek, Georgia, USA). Bead milling (5,000 rpm for 30 s, Precellys evolution homogenizer, Bertin Technologies, Montigny-le-Bretonneux, France) with 0.1 mm glass beads was used for cell lysis. The genomic DNA of *Sanguina nivaloides* was sequenced at low coverage by BGI (Guangdong, China) with 150 bp paired-end reads on a DNBSEQ instrument. The PhyloHerb genome skimming pipeline was used to assemble the full-length 18S + ITS1+5.8S + ITS2+28S rDNA sequence (70). For the rest of the taxa, 18S rDNA was amplified by polymerase chain reaction using universal primers NS1 and NS8 (71). The PCR products were purified and sequenced with Sanger sequencing at Macrogen (Amsterdam, Netherlands). The 18S sequences were aligned with MUSCLE (multiple sequence alignment by log-expectation) and trimmed to obtain the same lengths for all sequences (1,169 bp). Phylogeny of 18S rDNA sequences was constructed by Neighbor-joining in MEGAX 10.1.8 (72).

### Cell tracking under thermally controlled conditions

Cultures were incubated for 48 h at each temperature in 10 mL plastic flasks (T25 EasYFlask, ThermoFisher Scientific) inside the temperature-controlled incubator at 40 µmol⋅m^−2^⋅s^−1^ photons PAR. A volume of 5 µL of cell culture was loaded into a 20 µm-deep sperm counting chamber slide (CV 1020-2cv, CellVision technologies, The Netherlands) that was pre-incubated at the same temperature. The slide model was selected because it allows the enumeration of motile vs non-motile cells in the population, concurrent with tracking measurements of swimming cells. The loaded slide was immediately placed on a temperature-controlled microscope stage with thermoelectric cooling and heating accuracy ±0.1°C (PE120, Linkam, UK). Ten-second videos were recorded using an Olympus DP28 camera mounted on an Olympus BX-43 microscope (Olympus Europa GmbH, Hamburg, Germany) with 10 × magnification and 1,080 p/30fps resolution (imaging software cellSens Standard V3.2). The light intensity provided by the microscope white LED source (U-LHLEDC, 2 Watt) was also 40 µmol⋅m^−2^⋅s^−1^ photons PAR. For validation of the temperature control system and fast temperature change measurements, temperature programs of 5°C (constant low temperature), +2.5°C⋅min^−1^ (slow temperature ramp rising from 5°C), +10°C⋅min^−1^ (fast temperature ramp rising from 5°C), and Δ10°C⋅min^−1^ “thermal oscillation” between 5°C and 25°C were used (Fig. 1B through E).

### Video tracking analysis

Videos in .AVI file format were processed by first inverting the images using an X-Ray filter from iMovie (iMovie 10.4, Apple) and then converted into .TIFF image sequences (30 fps and 720 p). Image sequences were uploaded into Fiji ImageJ (73) in 16-bit as a virtual stack of image sequences. The TrackMate plugin (74) was then used, where cells visible as bright spots were detected with the DoG (Difference of Gaussian) detector and estimated object diameter of 10–12 pixels, depending on the species, using initial thresholding “Quality” of 0.67. A filter “Std intensity ch1” was slightly adjusted on each video batch to remove detected artifacts such as debris, and the “signal/Noise ratio ch1” filter was also used if needed. Track detection was performed using the LAP tracker, based on the “Linear Assignment Problem” mathematical framework with the following settings: a max distance of 30 pixels for frame-to-frame linking, a max distance of 50 pixels and 30 frames (1 s) for track segment gap closing. A filter “track duration” was then applied to remove any track shorter than 75 frames. “Spots” data files were exported for visualizing tracks and “Tracks” files for extracting movement parameters.

### Distinguishing motile vs non-motile cells

To distinguish motile cells actively swimming from non-motile cells that may be stationary or passively drifting, a threshold of total distanced traveled was defined based on measurements of control cells with their cilia removed (Fig. S7). Non-motile deciliated *C. reinhardtii* cells were prepared by applying a pH shock with 0.5 N acetic acid down to pH 4.5, followed by recovery with the addition of 0.5 M KOH up to pH 6.5 (75). Three technical replicates were loaded onto a sperm counting chamber, with a drop of culture intentionally left on the opening of the chamber to enhance the drifting of part of the population. Video tracking results are shown in Fig. S7, which demonstrate that the total distance traveled can be used to separate motile cells from stationary and passively drifting cells. A threshold of 100 µm was selected to distinguish the proportion of motile and non-motile cells, corresponding to an average speed of 10 µm⋅s^−1^. We note that this is lower than the value of 32 µm⋅s^−1^ used in another study on *Cd. reinhardtii* that also used a speed threshold to account for drifting (76). During the main experiment, over a thousand cells were tracked in each replicate, and the fraction of “motile” vs “non-motile” was determined for each replicate population. The means for the motility parameters, including swimming speed, were calculated based on the motile cell population of each replicate.

### Thermal performance curves of swimming speed

The effect of temperature on swimming speed was modeled with thermal performance curves using the rTPC package (77) in R (version 2023.12.1 + 402). A first selection of eight models with biological relevance for our experiment was fitted to data from all taxa (Fig. S8). Based on Akaike’s Information Criterion (AIC), as well as graphical suitability (Fig. S9), we considered three models: “Pawar,” “Thomas2,” and “Weibull.” The three models were fitted with a weighted non-linear regression using bootstrapping with residual resampling for calculating confidence intervals. One thousand generations were computed and weighted based on 1/sd. Based on results from the three models (Fig. S10), the “Pawar” model provided the best fit for *L. spitsbergensis* and *Gloeocystis* sp., while “Thomas2” model provided the best fit for the other seven species and was used to estimate the optimal temperature and associated maximum speed (*R*_max_) (37, 38).


(Pawar) $$rate=\;\frac{r_{tref}\cdot {exp}^{\frac{-e}{k}\;\left(\frac{1}{\;temp+273.15}\;-\;\frac{1}{t_{ref}+273.15}\;\right)}}{1+\left(\frac{e}{eh-e}\right)\cdot {exp}^{\frac{eh}{k}\;\left(\;\frac{1}{T_{opt}+273.15}\;-\;\frac{1}{temp+273.15}\right)\;}}$$

Here, temp is the temperature (°C), *t*_ref_ is the standardization temperature (temperature at which rates are not inactivated by high temperatures, in our case 1°C), *r*_tref_ is the rate at the standardized temperature, *e* is the activation energy (eV), *eh* is the high temperature activation energy (eV), *T*_opt_ is the optimum temperature (°C), and *K* is Boltzmann’s constant with a value of 8.62e^−5^.


(Thomas2) $$rate=a\cdot {exp}^{b-temp}-\left(c+d\cdot {exp}^{e-temp}\right)$$

Here, temp is the temperature (°C), *a* is the swimming speed at 0°C, *b* describes the exponential increase in swimming speed with increasing temperature, and *c* is the temperature independent inactivation term that attenuates swimming speed. Finally, *d* and *e* control the exponential increase in the inactivation of swimming speed as temperature rises, with *d* scaling this effect and *e* determining its sensitivity to temperature.

### Motility parameters

From the TrackMate data (73), in addition to speed (µm⋅s^−1^), we extracted “total distance traveled” (µm) and “maximum distance traveled” (µm) (Fig. S2A) and calculated the “confinement ratio” as “maximum distance traveled” divided by “total distance traveled.” Lower confinement ratios indicate cells remaining within a highly confined path. From the resulting confinement ratio, the “dispersal” (µm⋅s^−1^) was calculated as the swimming speed multiplied by the corresponding confinement ratio.

### Imaging flow cytometry

Cell morphology was measured in cultures treated at 5°C using an ImageStream^X^ MK II Imaging Flow Cytometer (Luminex Corporation, Austin, TX, USA), capturing a minimum of 5,000 objects and magnification of 60×. IDEAS v6.1.823.0 software was then used to process the recorded bright field pictures (Channel 4) by applying a mask covering the cell area and extracting measurements for the cell length, width, and area.

### Petri dish phototaxis assay

Cultures adjusted to an OD_680_ of 0.25 with fresh media were prepared (*n* = 6) and incubated for 2 days at a light intensity of 40 µmol⋅m^−2^⋅s^−1^ photons PAR. A temperature of 2.5°C was used throughout because it is close to the conditions found in melting snow. Prior to phototaxis experiments, each sample was dark acclimated for 1 h, and then 5 mL culture was transferred to a 35 mm Petri dish. The Petri dish was placed on a white LED panel for recording standardized pictures before and after migration. Phototaxis was induced by a 540 nm green LED at 40 µmol⋅m^−2^⋅s^−1^ photons PAR and placed at one side of the dish. The assay was run for 35 mins, allowing motile cells swimming >10 µm⋅s^−1^ to swim across a 35 mm Petri dish. The phototaxis index was determined as described in Wakabayashi et al. (65), by subtracting the empty Petri dish background image from images of the filled dish after the assay. Image analysis was done in ImageJ (Fiji) by inverting the color, converting to black and white, then recording white intensity values of the total Petri dish (total intensity) and the front half-dish (front intensity). The phototaxis index was calculated by the following equation:


$$Phototaxis\;index=\;\frac{\left(front\;intensity\;\;\times \;front\;area\right)}{\left(total\;intensity\;\times \;total\;area\;\right)}$$

Where the areas are in pixels. To determine for significant cell phototaxis, we tested whether the phototaxis index was significantly different from 0.5 using a *t*-test (*P* < 0.05, *n* = 6).

### Pulse amplitude modulation fluorometry

Photophysiology performance at each temperature was measured with a multi-color pulse amplitude modulation (PAM) fluorometer (Heinz Walz GmbH, Effeltrich, Germany) fitted with a temperature-controlled cuvette system. The instrument was set up with a Measuring Light of 1 (MF-L = 10, MF-H = 5000), Gain = 4 and Damping = 1. Saturation pulses were induced with 480 nm (blue) light of intensity of 12 and duration 600 ms recording the maximum quantum yield of photosystem II (Fv/Fm). Measurements were carried out in a 1 cm quartz cuvette containing dilute cell suspension of between 30 and 50 µL of culture in a total of 1,250 µL media enriched with 10 mM HCO_3_^−^. Filled cuvettes were dark acclimated for 30 min at study temperature before measurement. The corresponding swimming speed TPC models were also applied to the Fv/Fm data sets. The correlation between *T*_opt_ Fv/Fm and *T*_opt_ swimming speed was assessed by evaluating if the linear relationship differed significantly from a slope of 1 (CI-low < 1 < CI-high).

### Statistics

Linear mixed-effects regression models were fitted using the R package lme4 (version 1.1–35.5) (78) to the experimental benchmarking data (Fig. 1). For all “controls,” the model assessed the main effect of time on swimming speed, specifying random intercepts and slopes for each replicate (*n* = 3). For “temperature oscillation,” a linear model was applied to evaluate the effect of time on swimming speed at each temperature extreme or condition, i.e. 5°C or 25°C step of oscillation, but specifying only a random intercept as the most efficient, simpler model (Table S2).

Two sample *t*-tests were applied to compare TPC parameters between both temperature ramps in the experimental benchmarking (*n* = 3, Fig. 1), and one-sample *t*-tests were applied to phototaxis data to test whether the cell distribution across the dish was significantly different from 0.5 (*n* = 6, Fig. 2A).

R Studio (Version 2023.12.1+402) was used to generate all statistics and models in this study.

## Data Availability

Original data for this paper are available on the platform Zenodo at https://doi.org/10.5281/zenodo.11113321.

## SUPPLEMENTAL MATERIAL

Supplemental information mbio.02954-24-s0001.docx Supplemental figures, tables, and equation.

ASM does not own the copyrights to Supplemental Material that may be linked to, or accessed through, an article. The authors have granted ASM a non-exclusive, world-wide license to publish the Supplemental Material files. Please contact the corresponding author directly for reuse.
