# Supplementary material for: Snow algae exhibit diverse motile behaviors and thermal responses
Source: mBio. 2025 Apr 1;16(5):e02954-24. doi: 10.1128/mbio.02954-24 (PMC12077220; doi:10.1128/mbio.02954-24)
Supplement: Supplemental information — Supplemental figures, tables, and equation. [file mbio.02954-24-s0001.docx]

**Supplementary Information:**

**Snow algae exhibit diverse motile behaviours and thermal responses**

Alexandre Détain^1^*, Hirono Suzuki^1^, René H. Wijffels^1,2^, Nathalie Leborgne-Castel^3^, Chris J. Hulatt^1^*

^1^Faculty of Biosciences and Aquaculture, Nord University, Bodø, Norway

^2^Bioprocess Engineering, AlgaePARC, Wageningen University, Wageningen, the Netherlands

^3^Agroécologie, INRAE, Institut Agro, Université Bourgogne Europe, Dijon, France

*e-mail: [alexandre.detain@nord.no](mailto:alexandre.detain@nord.no); [christopher.j.hulatt@nord.no](mailto:christopher.j.hulatt@nord.no)


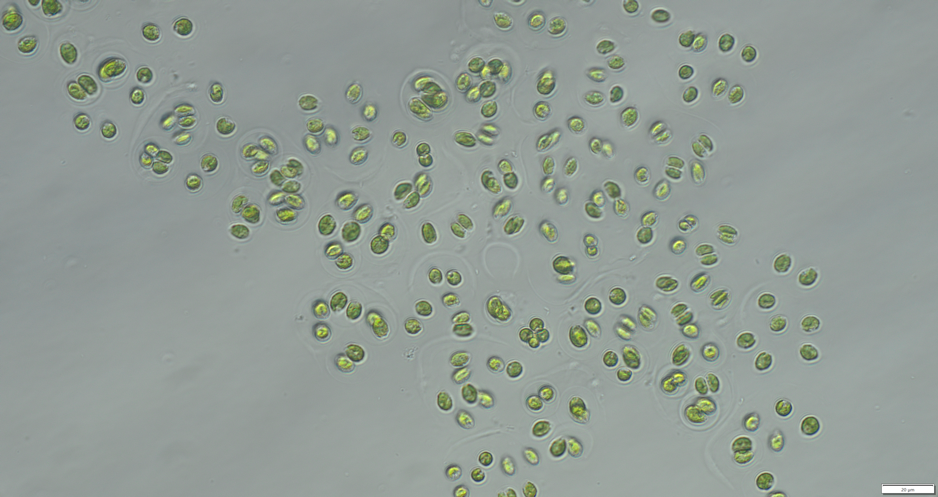

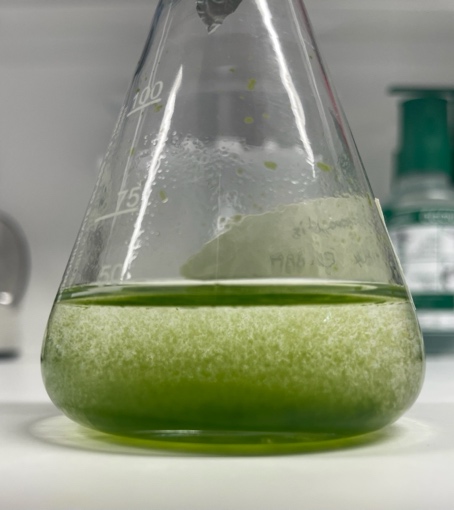


**Fig. S1 *Gloeocystis* sp. culture with mucilage accumulation.** *Left*, microscope picture of *Gloeocystis* sp. culture with cells accumulating mucilage around (black arrows), the scale bar indicates 20µm. *Right*, Erlenmeyer flask of *Gloeocystis* sp. culture, with aggregates/clouds of cells slowly sinking down in the “viscous” medium.


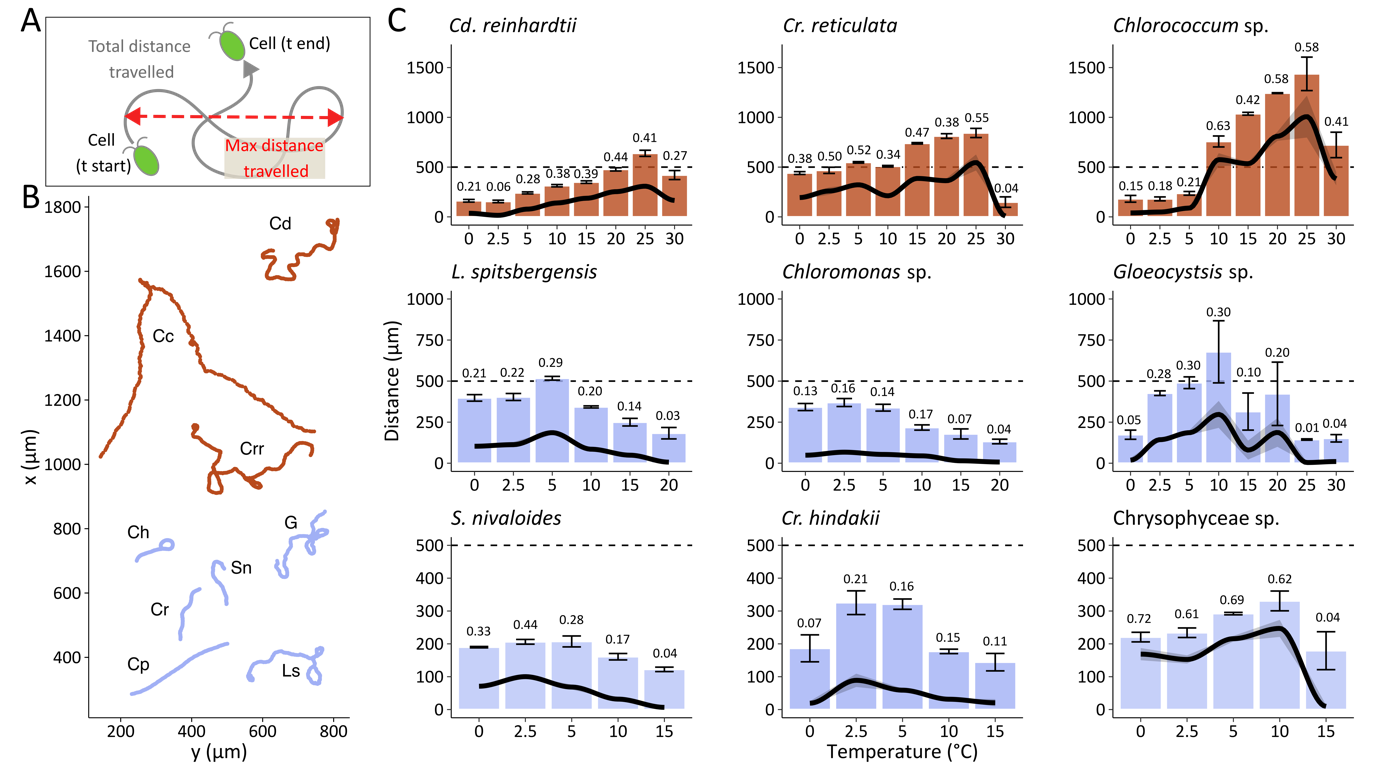


**Fig. S2 Displacement of species over 10s.** (**A**) Definition of the ‘Max distance travelled’ in comparison to the ‘Total distance travelled’, which correspond to the maximum distance between the two furthest points of a measured track. (**B**) Representative 10s tracks for each species; tracks were selected based on their mean speed and confinement ratio values matching calculated averages from the **Fig. S5**. *Cd:* *Cd. reinhardtii, Ls: L. spitsbergensis, Sn: S. nivaloides, G: Gloeocystis* sp*., Cc: Chlorococcum* sp*., Crr: Cr. reticulata, Ch: Cr. hindakii, Cr: Chloromonas* sp*., Cp:* Chrysophyceae sp.. (**C**) Means (±sd, n=3) of Total distance travelled (bar chart) with corresponding means (±sd, n = 3, shaded area) of Max distance travelled (black line) and calculated average confinement ratio (digits, **Table S1**) over temperature (°C). Dashed lines mark 500 µm for all plots. Blue and brown colour code differentiates taxa with cryophilic behaviour (blue) from cryotolerant/mesophilic behaviour (brown) observed in this study.


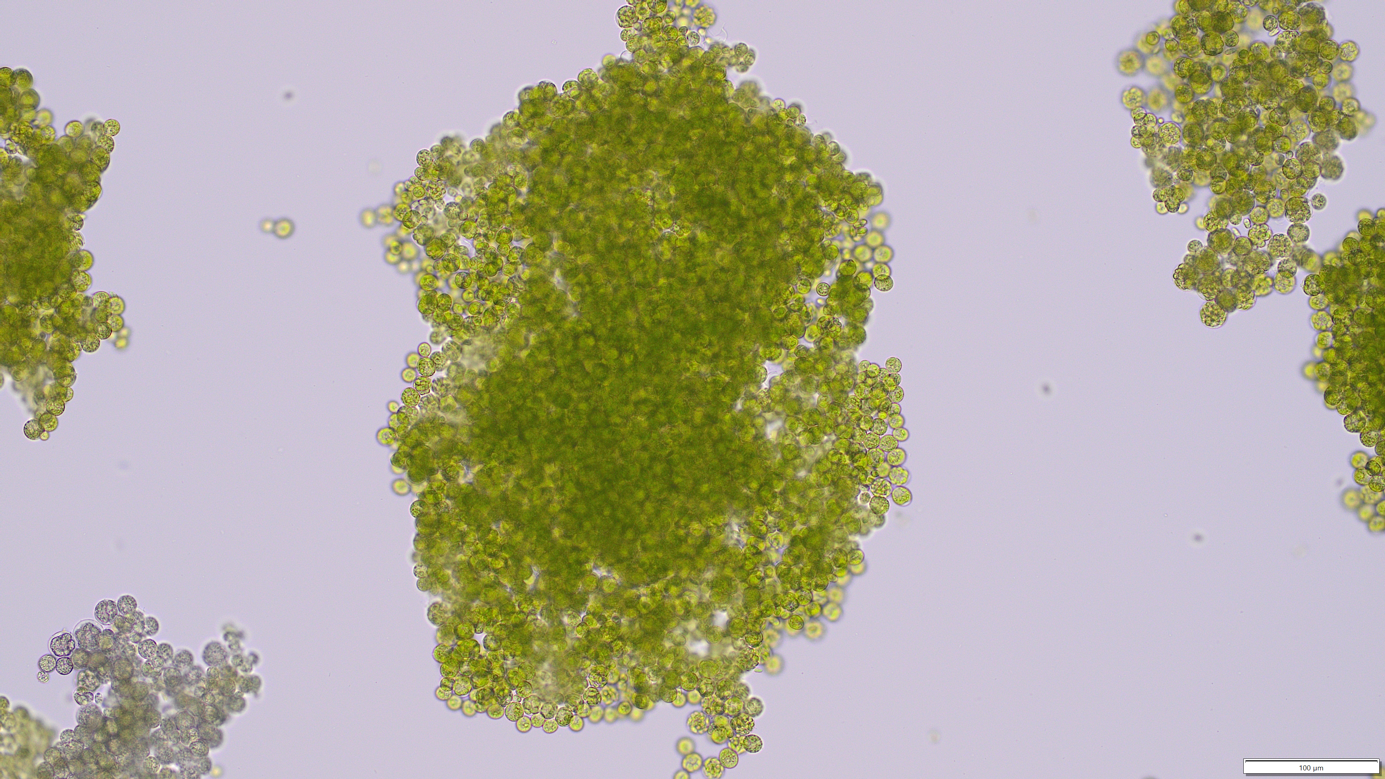


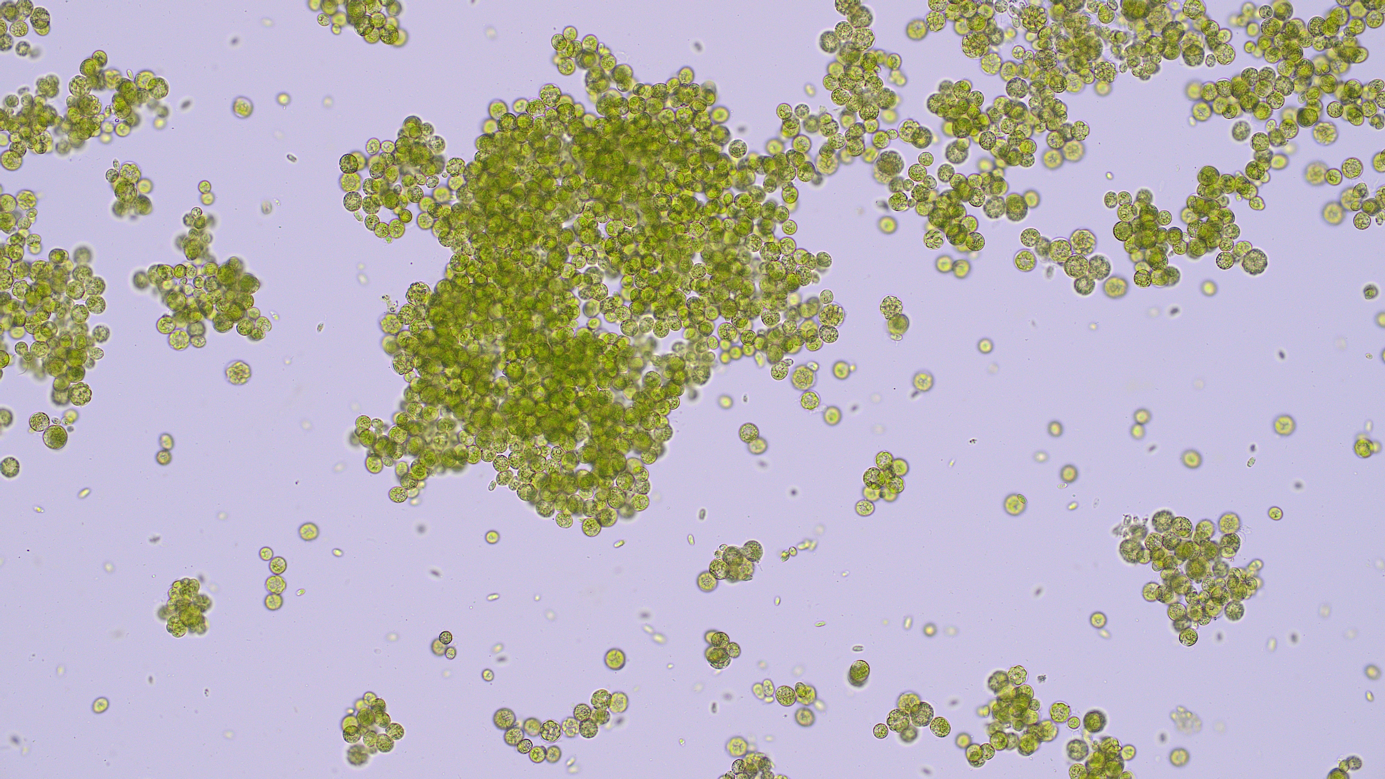


**Fig. S3 *Chlorocccum* sp*.* (ARK-S22-20) cell morphology and behaviour at 0°C.** Aggregates of cells (upper), and the same aggregate partially burst after the microscope cover slide was squished on the slide (bottom). Small swimming cells, of which representatives are indicated by black arrows, were released from the aggregates. The large, immobile cells and hiding behaviour of small motile biciliate cells was unique to this strain at low temperature (DOI 10.5281/zenodo.11113321, before: Movie_3184.avi; after: Movie_3185).


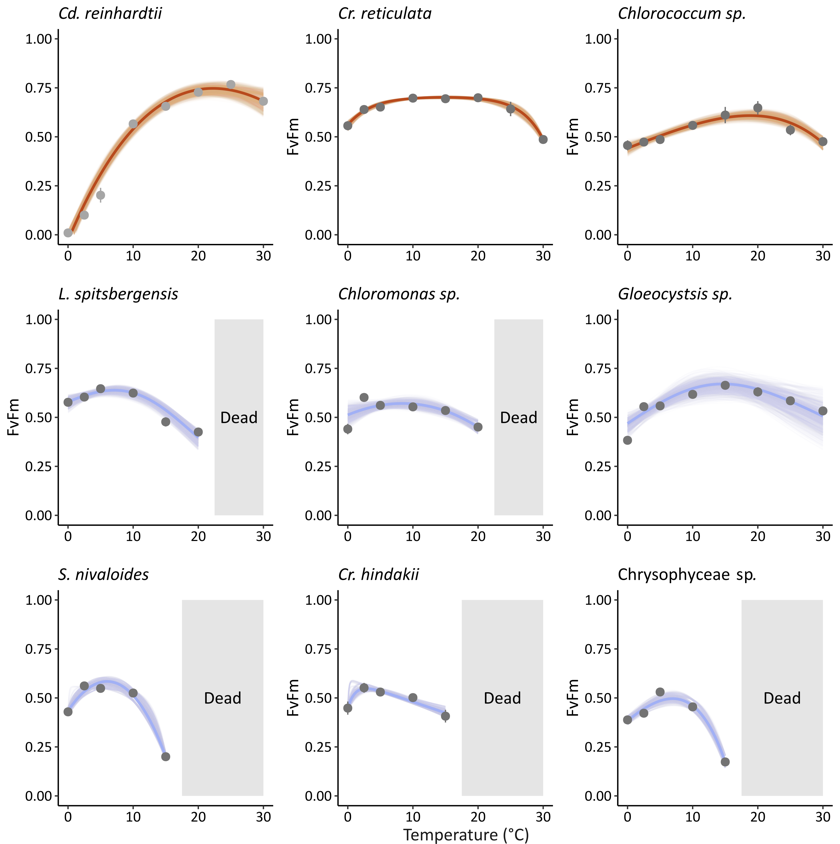


**Fig. S4 Thermal performance curves of the maximum quantum yield of photosystem II (Fv/Fm).** TPCs were fitted with the same method as for swimming speed (i.e. using the weighted bootstrapping method, coloured lines) on the means of biological replicates (n = 3, grey dots). When no chlorophyll fluorescence was measured at high temperature, a value of 0 was attributed and omitted from the thermal performance curve modelling.


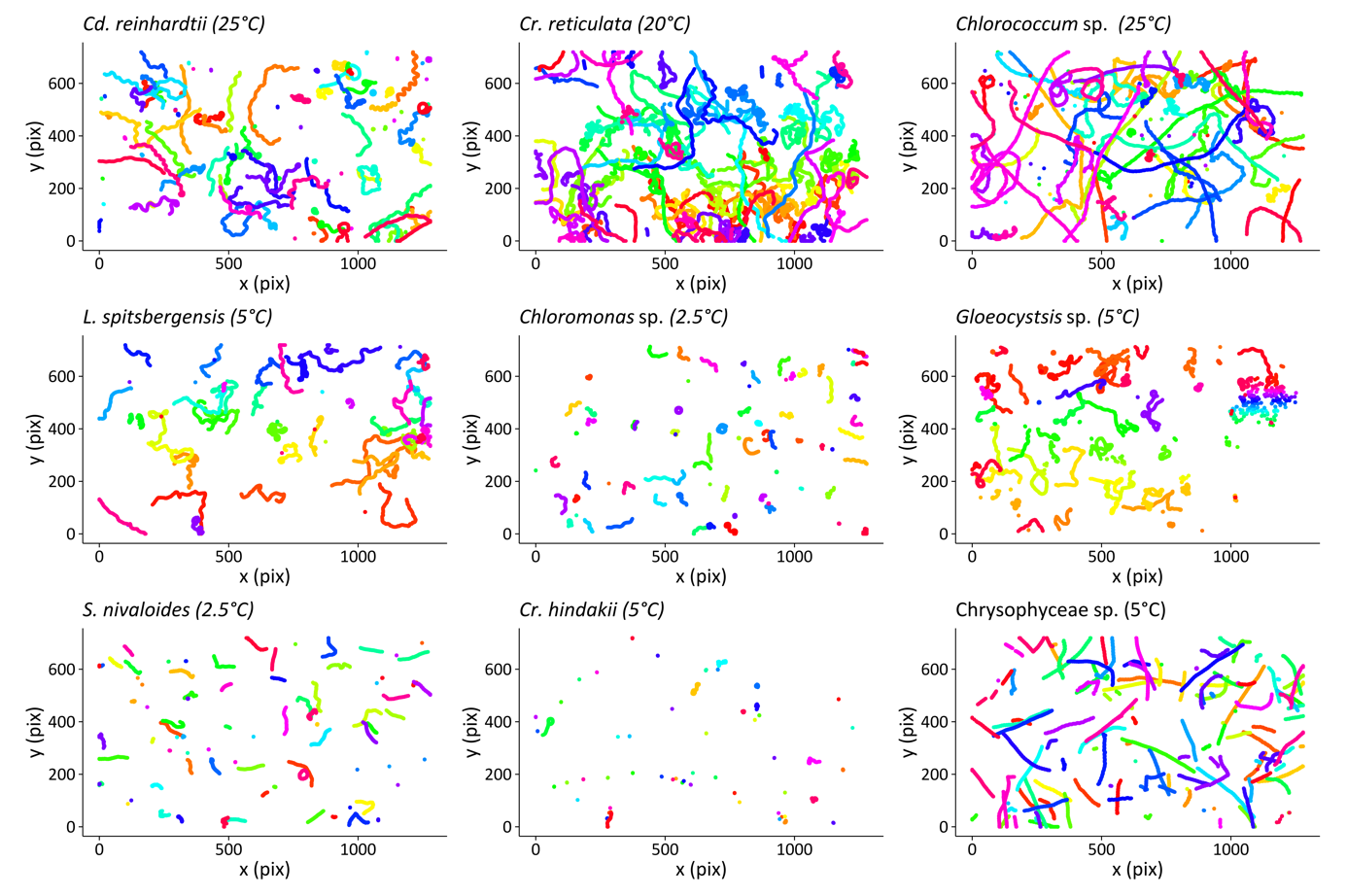


**Fig. S5. Example tracks measured from a selected video of each species.** Projections of tracks are displayed on an x/y axis in pixels (pix). Videos were randomly selected from the pool of the closest optimal temperature defined by TPCs (Fig. 1). 5°C was chosen as optimal for Chrysophyceae sp. since the motile proportion was higher at 5°C.


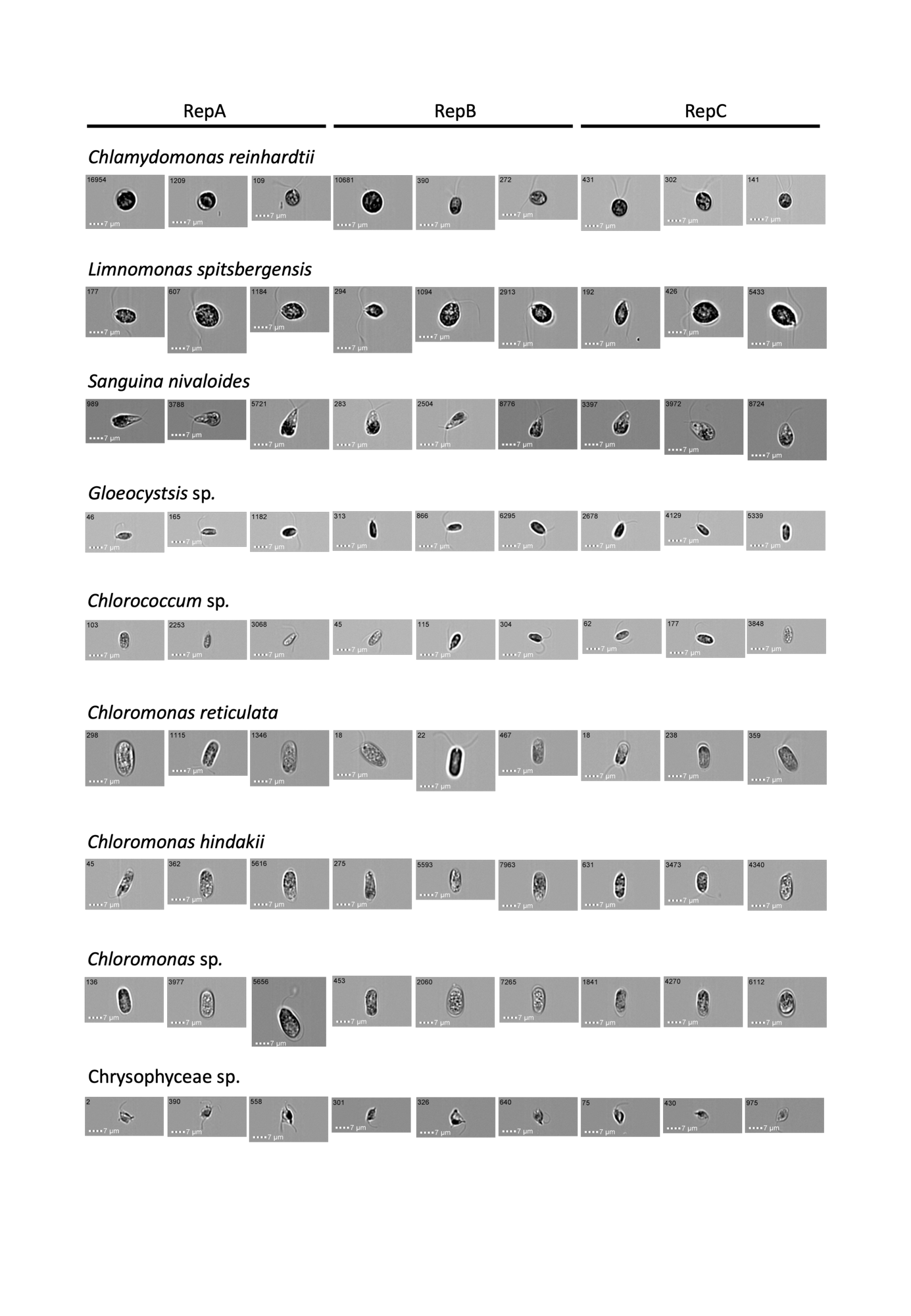


**Fig. S6. Selected brightfield images of the motile cells of the 9 species from imaging flow cytometry.** Three images are presented per biological replicate (n=3).


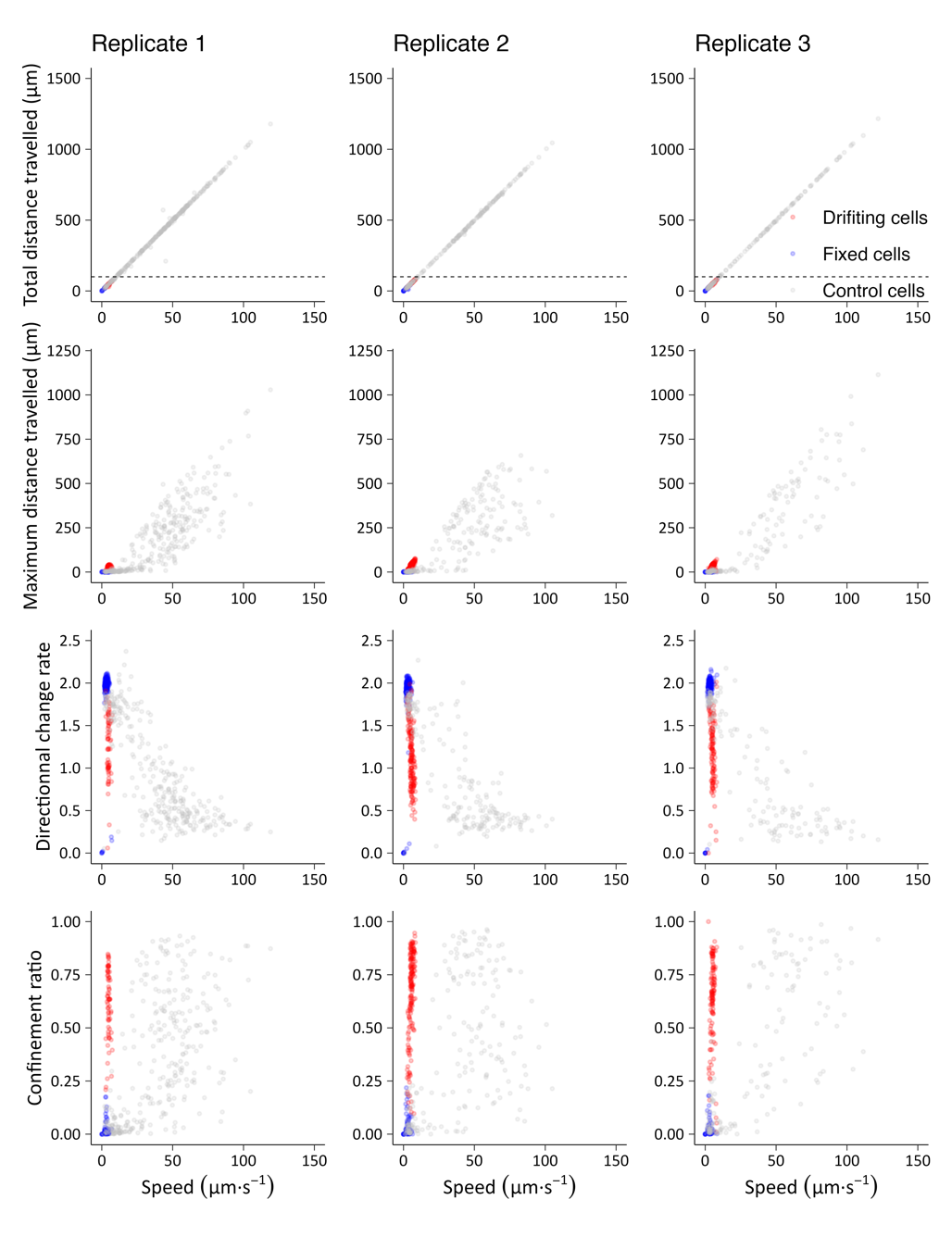


**Fig. S7. Swimming speed correlated with other measured parameters for drifting, fixed, and control *Chlamydomonas reinhardtii* populations.** This analysis was conducted to determine the identification of actively swimming cells from those passively drifting. Three technical replicates were tested. The panel shows that drifting cells (red) can overlap with other motile cells for the ‘confinement ratio’ and ‘directional change’. It also overlaps with a small proportion of motile cells (cells swimming up to about 50 µm⋅s^-1^) for ‘Maximum distance travelled’. Only the parameter ‘Total distance travelled’ did not overlap with the motile cell population, however this feature is directly proportional to the speed and is calculated from it. According to these results, to ensure identification of fixed and drifting cells in the motile population, 100 µm of total distance travelled (dashed line) was used as the threshold for defining motile and non-motile cell populations.


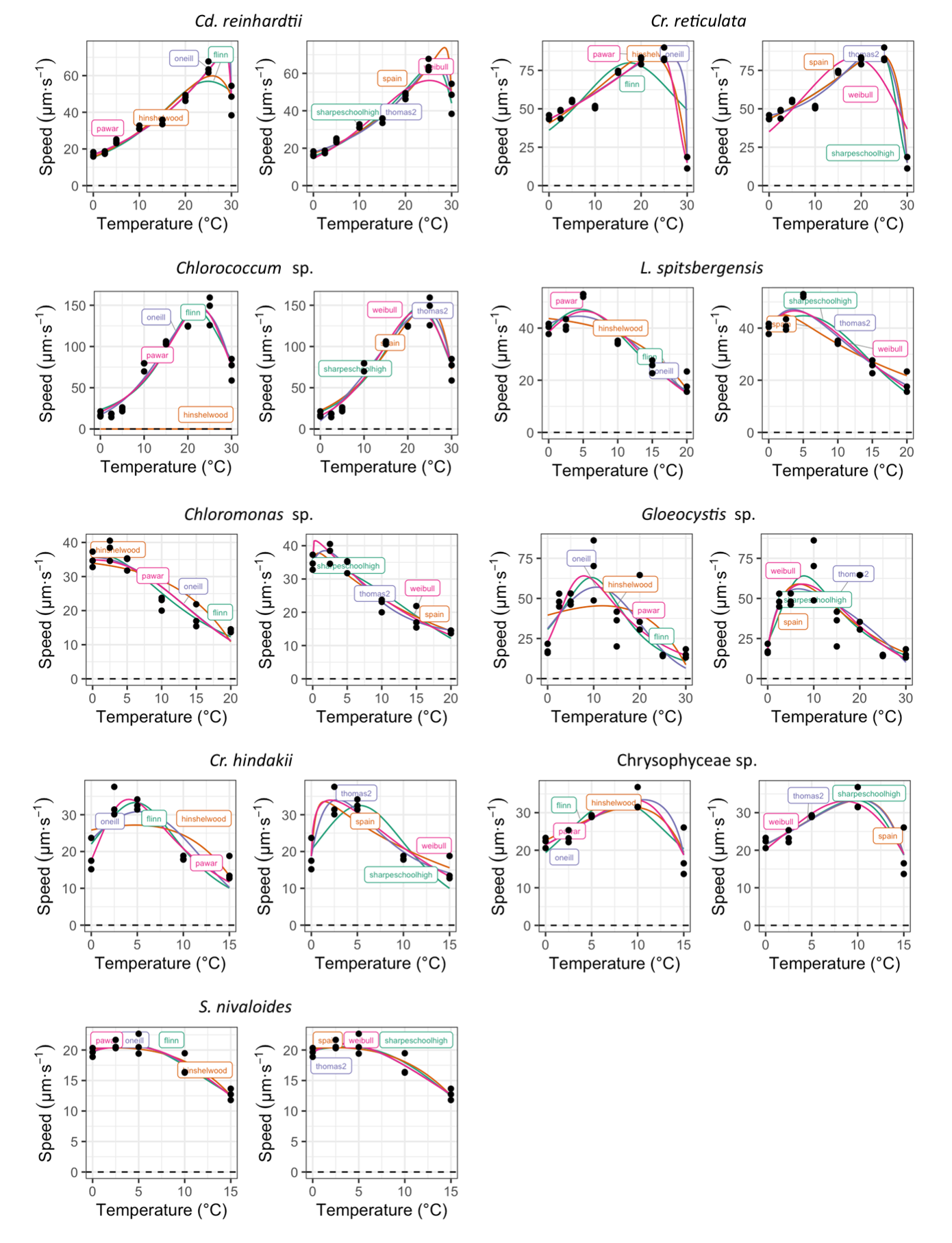


**Fig. S8 Comparison of Thermal Performance Curve models with rTPC for the nine studied species.** Pre-selected models (Hinshelwood, Finn, Oneil, Pawar, Sharpeschoolhigh, Spain, Thomas2 and Weibull) from the rTPC package were fitted to assess how they describe speed the temperature-dependence of swimming speed.

| **Table of AIC score per species** | | | | | | | | | | |
| --- | --- | --- | --- | --- | --- | --- | --- | --- | --- | --- |
| Model | *Cd. reinhardtii* | *L. spitsbergensis* | Chrysophyceae sp. | *Cr. reticulata* | *Chloromonas* sp. | *Cr. hindakii* | *Gloeocystis* sp. | *Chlorococcum* sp. | *S. nivaloides* | sum |
| Flinn | 151 | 108 | 86,6 | 195 | 93,7 | 93 | 199 | 204 | 51,5 | 1181,8 |
| Oneil | 133 | 117 | 83,8 | 145 | 103 | 101 | 203 | 192 | 54,3 | 1132,1 |
| Pawar | 133 | 113 | 83,7 | 143 | 105 | 88,7 | 197 | 198 | 54,9 | 1116,3 |
| Sharpesch. | 140 | 116 | 83,7 | 143 | 102 | 97,7 | 197 | 198 | 55,5 | 1132,9 |
| Spain | 133 | 120 | 83,7 | 143 | 95 | 90,4 | 197 | 197 | 57,3 | 1116,4 |
| Thomas2 | 136 | 114 | 85,7 | 143 | 88,4 | 85,7 | 200 | 190 | 55,6 | 1098,4 |
| Weibull | 155 | 112 | 85,5 | 189 | 89,8 | 86,8 | 197 | 194 | 53,6 | 1162,7 |
|  |  |  |  |  |  |  |  |  |  |  |
| **Table of AIC rank by species** | | | | | | | | | | |
| Model | *Cd. reinhardtii* | *L. spitsbergensis* | Chrysophyceae sp. | *Cr. reticulata* | *Chloromonas* sp. | *Cr. hindakii* | *Gloeocystis* sp. | *Chlorococcum* sp. | *S. nivaloides* | sum |
| Flinn | 4 | 1 | 5 | 4 | 3 | 5 | 3 | 7 | 1 | 33 |
| Oneil | 1 | 6 | 2 | 2 | 6 | 7 | 5 | 2 | 3 | 34 |
| Pawar | 1 | 3 | 1 | 1 | 7 | 3 | 1 | 5 | 4 | 26 |
| Sharpesch. | 3 | 5 | 1 | 1 | 5 | 6 | 1 | 5 | 5 | 32 |
| Spain | 1 | 7 | 1 | 1 | 4 | 4 | 1 | 4 | 7 | 30 |
| Thomas2 | 2 | 4 | 4 | 1 | 1 | 1 | 4 | 1 | 6 | 24 |
| Weibull | 5 | 2 | 3 | 3 | 2 | 2 | 2 | 3 | 2 | 24 |
|  |  |  |  |  |  |  |  |  |  |  |
| **Table rank by species** | | | | | | | | | | |
| Model | *Cd. reinhardtii* | *L. spitsbergensis* | Chrysophyceae sp. | *Cr. reticulata* | *Chloromonas* sp. | *Cr. hindakii* | *Gloeocystis* sp. | *Chlorococcum* sp. | *S. nivaloides* |  |
| Flinn |  |  |  |  |  |  |  |  |  |  |
| Oneil |  |  |  |  |  |  |  |  |  |  |
| Pawar |  |  |  |  |  |  |  |  |  |  |
| Sharpesch. |  |  |  |  |  |  |  |  |  |  |
| Spain |  |  |  |  |  |  |  |  |  |  |
| Thomas2 |  |  |  |  |  |  |  |  |  |  |
| Weibull |  |  |  |  |  |  |  |  |  |  |

**Fig. S9 Comparison of thermal performance curve models with rTPC for the 9 studied species.** The first table represents Akaike information criterion (AIC) scores, where the lower score indicates a more efficient model. The second table shows how each model ranks in AIC score for each species. The last *sum* column of the first two tables respectively ‘*sum’* of AIC score and ‘rank’ of AIC score to evaluate which model score, on average, is the best across all species. Heat-map colour from blue (lowest) to red (highest) is used for both of these tables. The last table reports results from graphical curve fit suitability, with green as suitable, and red as unsuitable based on the T_opt_ estimation and capacity to represent measured data and provide the closest conclusion to observations.


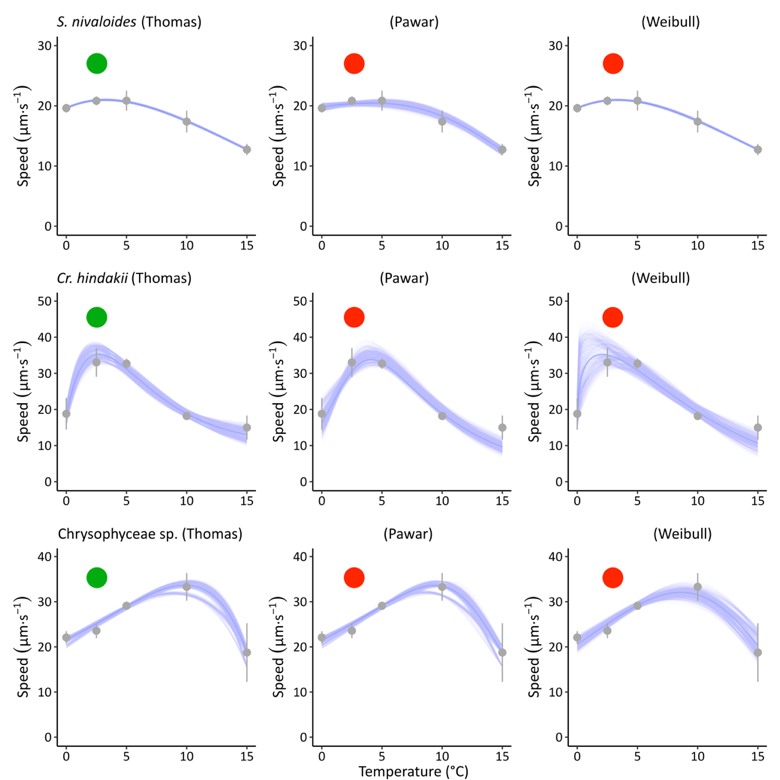

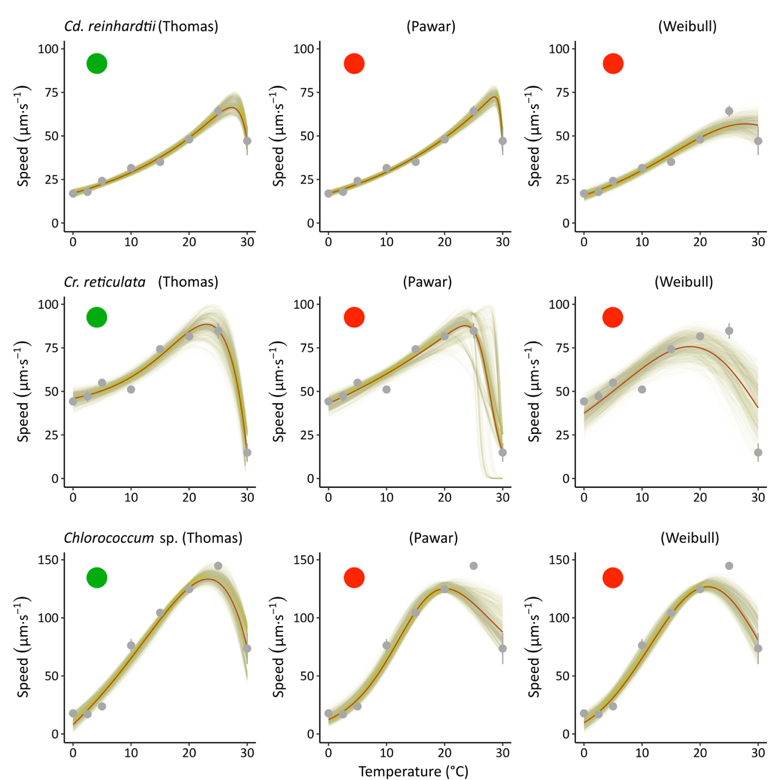
**
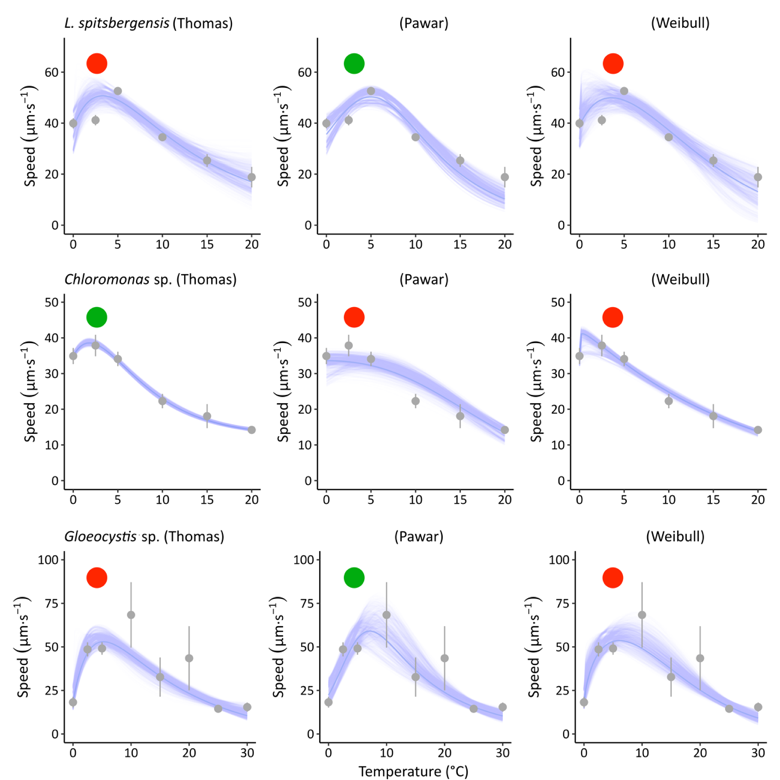
**

**Fig. S10 Comparison of three best thermal performance curve models with rTPC for the 9 studied species.** Grey dots represent mean (±sd, n = 3) of measured swimming speed. The three best models (Thomas2 or Thomas, Pawar and Weibull) were selected based on their AIC and graphical suitability. Models were fitted using residual resampling weighted non-linear regression, and the best graphically suitable model with a green mark was selected for the rest of the study. Note that Thomas model was selected for all species except for *L. spitsbergensis* and *Gloeocystis* sp. where Pawar was used.

**Equation S1. The Weibull model equation used to fit thermal response curves in Supplementary Fig. 4.**

$$rate=a \times\left( \frac{c-1}{c} \right)^{\frac{1-c}{c}}\times\left( \frac{temp-T_{opt}}{b}+\left( \frac{c-1}{c} \right)^{\frac{1}{c}} \right)^{c-1}\times{exp}^{\left( \frac{temp-T_{opt}}{b}+\left( \frac{c-1}{c} \right)^{\frac{1}{c}} \right)^{c}}+\frac{c-1}{c}$$

Here, *temp* is the temperature (°C), and T_opt_ the optimum temperature for the swimming speed (*rate*). The parameter *a* defines the height, *b* the breadth and *c* the shape of the curve.

Reference: Angilletta Jr, Michael J. Estimating and comparing thermal performance curves. Journal of Thermal Biology 31.7 (2006): 541-545.

**Table S1.** Confinement ratio means ±sd (n = 3) for each species and temperature, calculated from ‘Max distance travelled’ divided by ‘Total distance travelled’. (-) indicates no data measured.

| **Species** | **0°C** | **2.5°C** | **5°C** | **10°C** | **15°C** | **20°C** | **25°C** | **30°C** |
| --- | --- | --- | --- | --- | --- | --- | --- | --- |
| *Limnomonas spitsbergensis* | 0.24  ±0.02 | 0.26  ±0.03 | 0.33  ±0.01 | 0.23  ±0.01 | 0.18  ±0.01 | 0.04  ±0.03 | - | - |
| *Chlamydomonas reinhardtii* | 0.23  ±0.03 | 0.09  ±0.06 | 0.30  ±0.04 | 0.41  ±0.06 | 0.41  ±0.01 | 0.46  ±0.02 | 0.44  ±0.02 | 0.30  ±0.02 |
| *Sanquina nivaloides* | 0.35  ±0.01 | 0.45  ±0.01 | 0.29  ±0.02 | 0.19  ±0.02 | 0.05  ±0.01 | - | - | - |
| *Gloeocystis* sp*.* | 0.07  ±0.04 | 0.33  ±0.01 | 0.34  ±0.02 | 0.35  ±0.04 | 0.14  ±0.06 | 0.21  ±0.04 | 0.03  ±0.02 | 0.06  ±0.06 |
| *Chlorococcum* sp*.* | 0.17  ±0.08 | 0.23  ±0.03 | 0.24  ±0.01 | 0.66  ±0.03 | 0.46  ±0 .01 | 0.62  ±0.04 | 0.61  ±0.08 | 0.44  ±0.04 |
| *Chloromonas reticulata* | 0.40  ±0.03 | 0.52  ±0.06 | 0.55  ±0.02 | 0.38  ±0.02 | 0.51  ±0.02 | 0.42  ±0.02 | 0.57  ±0.07 | 0.06  ±0.01 |
| *Chloromonas hindakii* | 0.10  ±0.03 | 0.23  ±0.04 | 0.18  ±0.02 | 0.17  ±0.01 | 0.13  ±0.08 | - | - | - |
| *Chloromonas* sp*.* | 0.14  ±0.02 | 0.18  ±0.01 | 0.16  ±0.02 | 0.19  ±0.02 | 0.09  ±0.01 | 0.05  ±0.01 | - | - |
| Chrysophyceae sp. | 0.73  ±0.03 | 0.62  ±0.04 | 0.71  ±0.04 | 0.64  ±0.04 | 0.05  ±0.02 | - | - | - |

**Table S2.** ANOVA comparison of linear mixed-effects models on thermal oscillation benchmarking data. *Top*, presentation of models. *Bottom*, results from ANOVA between model 1 and 2 for *Cr. reticulata* and *L. spitsbergensis*. No-significant difference (ns) between models was approved with a P > 0.05.

**Models**

| model1: | speed ~ time * condition + (1 \| replicate) | | | | | | | |
| --- | --- | --- | --- | --- | --- | --- | --- | --- |
| model2: | speed ~ time * condition + (0 + time \| replicate) + (1 \| replicate) | | | | | | | |
|  | | | | | | | | |
| ***Cr. reticulata*** | npar | AIC | BIC | logLik | deviance | Chisq | Df | Pr(>Chisq) |
| model1 | 6 | 142.92 | 151.90 | -65.46 | 130.92 |  |  |  |
| model2 | 7 | 144.92 | 155.39 | -65.46 | 130.92 | 0 | 1 | 1 (ns) |

***L.***

***spitsbergensis***

| model1 | 6 | 199.67 | 208.65 | -93.83 | 187.67 |  |  |  |
| --- | --- | --- | --- | --- | --- | --- | --- | --- |
| model2 | 7 | 198.45 | 208.93 | -92.23 | 184.45 | 3.2169 | 1 | 0.07 (ns) |
